# Functional genetic variants of *GEN1* predict overall survival of Chinese epithelial ovarian cancer patients

**DOI:** 10.1186/s12967-024-05236-1

**Published:** 2024-06-18

**Authors:** Haoran Li, Jiao Wu, Qing Xu, Yangyang Pang, Yanzi Gu, Mengyun Wang, Xi Cheng

**Affiliations:** 1 Department of Gynecological Oncology, Fudan University Shanghai Cancer Center, Shanghai, China; 2 Department of Oncology, Shanghai Medical College, Fudan University, 270 Dong’an Road, Shanghai, 200032 China; 3 Cancer Institute, Fudan University Shanghai Cancer Center, 270 Dong’an Road, Shanghai, 200032 China; 4 Department of Gynecological Oncology, Minhang Branch, Fudan University Shanghai Cancer Center, Shanghai, China; 5 Department of Urology, Shanghai Xuhui Central Hospital, Zhongshan-Xuhui Hospital, Fudan University, Shanghai, China; 6 Department of Biobank, Fudan University Shanghai Cancer Center, Shanghai, China

**Keywords:** Genetic variant, Single nucleotide polymorphisms, Epithelial ovarian cancer, DNA double strand break repair, *GEN1*, Overall survival

## Abstract

**Background:**

Inherited variations in DNA double-strand break (DSB) repair pathway are known to influence ovarian cancer occurrence, progression and treatment response. Despite its significance, survival-associated genetic variants within the DSB pathway remain underexplored.

**Methods:**

In the present study, we performed a two-phase analysis of 19,290 single-nucleotide polymorphisms (SNPs) in 199 genes in the DSB repair pathway from a genome-wide association study (GWAS) dataset and explored their associations with overall survival (OS) in 1039 Han Chinese epithelial ovarian carcinoma (EOC) patients. After utilizing multivariate Cox regression analysis with bayesian false-discovery probability for multiple test correction, significant genetic variations were identified and subsequently underwent functional prediction and validation.

**Results:**

We discovered a significant association between poor overall survival and the functional variant *GEN1* rs56070363 C > T (CT + TT vs. TT, adjusted hazard ratio (HR) = 2.50, P < 0.001). And the impact of *GEN1* rs56070363 C > T on survival was attributed to its reduced binding affinity to hsa-miR-1287-5p and the resultant upregulation of *GEN1* mRNA expression. Overexpression of *GEN1* aggregated EOC cell proliferation, invasion and migration presumably by influencing the expression of immune inhibitory factors, thereby elevating the proportion of polymorphonuclear myeloid-derived suppressor cells (PMN-MDSCs) and then constructing an immunosuppressive tumor microenvironment.

**Conclusions:**

In conclusion, *GEN1* rs56070363 variant could serve as a potential predictive biomarker and chemotherapeutic target for improving the survival of EOC patients.

**Supplementary Information:**

The online version contains supplementary material available at 10.1186/s12967-024-05236-1.

## Highlights

Inherited variations in DSB repair pathway are known to influence ovarian cancer occurrence, progression and treatment response. In the present study, we performed a two-phase analysis of 19,290 SNPs in 199 genes in the DSB repair pathway among 1,039 ovarian cancer patients. Ultimately, we identified that the poor overall survival was significantly associated with functional variant *GEN1* rs56070363 C > T. Further investigations revealed the mechanism underlying this association: the C > T transition resulted in decreased binding affinity to hsa-miR-1287-5p and subsequent upregulation of *GEN1* mRNA expression. Overexpression of *GEN1* aggregated EOC cell proliferation, invasion and migration presumably by influencing the expression immune inhibitory factors, thereby elevating the proportion of PMN-MDSCs and then constructing an immunosuppressive tumor microenvironment. Our findings thus provide novel molecular targets and a theoretical basis for individualized treatment approaches in ovarian cancer.

**Supplementary Information:**

The online version contains supplementary material available at 10.1186/s12967-024-05236-1.

## Introduction

Epithelial ovarian carcinoma (EOC), the predominant histologic subtype of ovarian cancer, is presented as the leading cause of mortality among malignancies of the female reproductive system. Around 55% of EOC patients have already been at an advanced stage when diagnosed. For advanced EOC, the five-year overall survival rate remains poor [1]. Lack of early diagnostic methods and a high incidence of relapse due to the chemoresistance onset especially to the platinum-derived chemotherapeutics are the main reasons for the primary challenges in EOC management [2]. Given these critical issues, EOC presents as a formidable oncological challenge, necessitating the identification of novel prognostic biomarkers and the development of innovative therapeutic strategies to augment survival outcomes.

Human cells undergo around 70,000 DNA damage every day, and it is the DNA damage repair (DDR) pathway that maintains the genomic integrity. Increasing studies suggest that dysfunctions in the DDR pathway are critical factors influencing cancer occurrence, progression and treatment response [3]. Aberrations of DDR pathway may be manifested in the occurrence of genetic variants especially single nucleotide polymorphisms (SNPs). SNPs in DDR pathway genes can lead to alternations in protein function and DNA repair efficiency, resulting in varying prognostic outcomes for ovarian cancer patients.

To date, only a limited number of studies have identified SNPs in DNA damage repair genes, delineating their implications in the risk [4], prognosis [5]and chemotherapy response [6] of ovarian cancer. Hence, the biological significance of SNPs of other genes in DDR pathway needs to be explored. Our previous research focused on assessing the active involvement of potentially functional genetic variants in the nucleotide excision repair (NER) pathway and their impact on the response to platinum-based treatments [7]. In the current study, we intended to investigate SNPs in another DDR pathway, specifically the DNA double-strand break (DSB) repair pathway. DSB are among the most lethal damages and should be systematically repaired to maintain the stability of the genome. Consequently, SNPs in this pathway are likely to be of critical biological significance. We performed a two-phase analysis of 19,290 SNPs in 199 genes in the DSB pathway from a genome-wide association study (GWAS) dataset and explored their associations with overall survival (OS) in 1,039 Han Chinese EOC patients.

Ultimately, we selected *GEN1* rs56070363 for survival analysis and conducted both functional prediction and validation of this significant SNP. *GEN1* plays a pivotal role in the homologous recombination process, facilitating the repair of DSB. Acting as a human Holliday junction resolvase, *GEN1* functions efficiently to eliminate persistent recombination intermediates that hinder proper chromosome segregation during anaphase [8]. Therefore, *GEN1* is essential for maintaining genome stability and proper chromosome segregation [9]. So far, the role of *GEN1* in the ovarian cancer remained unclear. In addition, the mechanism by which common variants of *GEN1* contribute to the increased mortality risk in Chinese ovarian cancer patients remains to be elucidated. Previous findings suggested that *GEN1* rs56070363 influenced *GEN1* expression. We hypothesized the variants of *GEN1* influenced allele-specific microRNA binding which affected GEN1 expression, ovarian cancer cell function and tumor microenvironment.

## Materials and methods

### Patients selection

A total of 1039 patients enrolled in the present study were unrelated ethnic Han Chinese women with histologically confirmed EOC between August 2012 and January 2016. Blood samples were collected for the purpose of research by the tissue bank of Fudan University Shanghai Cancer Center (FUSCC) and the genotype data were generated as we previously reported [7]. Patients were randomly divided into two groups: a discovery group (n = 519) and a validation group (n = 520). The written informed consents were obtained from all recruited patients. The present study was approved by Ethics Committee at FUSCC (Approval no.050432–4-1911D) and conducted according to the principles in the Declaration of Helsinki consent.

### Data collection

Clinicophathological characteristics, including age at diagnosis, International Federation of Gynecology and Obstetrics (FIGO) stage, histology, grade, residue (optimal debulking < 1 cm), neoadjuvant chemotherapy information and chemotherapeutic response were collected. Primary patients with an early stage (FIGO stage I and II) tumor received complete staging surgery, while patients with a late stage (FIGO stage III and IV) tumor underwent cytoreductive surgery. After primary surgery, if necessary, some patients received platinum-based chemotherapies. Then, when the treatment was completed, patients were followed up every three months for the first two years, every six months for the next three years, and annually for the following years thereafter. OS were calculated from the date of first surgery to either the date of cancer-related death, or the last recorded visit.

### SNP selection, genotyping and quality control

Genes from the DSB repair pathway were screened out by the keyword “DNA double strand break repair” in the Molecular Signatures Database (http://software.broadinstitute.org/gsea/msigdb/index.jsp) and “PathCards” (http://pathcards.genecards.org/). After filtering out the duplicated genes and genes on the X chromosome, 199 genes remained as the candidate genes for further analysis. Genotyping data were obtained from a published GWAS datasets generated by Illumina Infinium Global Screening Array [10]. All SNPs in DSB pathway genes were extracted using Plink (version 1.09) (https://www.pngu.mgh.harvard.edu/purcell/plink/) [11]. Systematic quality control (QC) was applied to the raw genotyping data before the analysis, and the exclusion criteria were as follows: (1) with call rate less than 95%; (2) with map to X or Y chromosomes; (3) with MAF < 0.01; and (4) with Hardy–Weinberg equilibrium *P* < 1 × 10^–5^. The principal component analysis (PCA) was performed, and subsequent statistical analyses were adjusted for principal components that were related with OS of patients.

### Imputation

Additional SNPs of DSB pathway genes were imputed using IMPUTE 2.0 (https://mathgen.stats.ox.ac.uk/impute/impute_v2.html) with a linkage disequilibrium (LD) score of 0.8. Genotypes from the 1000 Genomes Phase 3 database were used as the reference data for imputation. QC was then performed for imputed genotypes by excluding SNPs with the following criteria: a posterior probability < 0.9, minor allele frequency (MAF) < 1%, missing genotypes > 5% or significant deviations from the Hardy–Weinberg equilibrium.

### Bayesian false-discovery probability

Since most of the SNPs in the present study were in LD as a result of imputation, we calculated Bayesian false-discovery probability (BFDP) as recommended [12] to assess the probability of false discoveries. In brief, the factors that could account for the value of BFDP include an estimate of the log relative risk, the variance of this estimate, the prior variance and the prior probability of a non-null association. For all selected SNPs, a threshold of BFDP value less than 0.8 was considered statistically noteworthy.

### Screening of relevant SNPs

To identify specific SNPs associated with OS, we used GenABEL package of R [13] to perform Cox regression analyses of OS, with adjustment for clinicopathological characteristics, including age, stage, histology, grade, residue, ascites and neoadjuvant chemotherapy. Meta-analysis was performed to combine the results of both discovery and validation datasets. A fixed-effects model was applied when the Cochran's Q-test *P* > 0.05 and the heterogeneity statistic (*I*^2^) < 50%. Otherwise, a random-effects model was employed. Receiver operating characteristic (ROC) curve was used to estimate the predictive value of genetic variants combined with clinical variables in additive models. To illustrate the fit of the model, an area under the curve (AUC) of ROC curves was calculated.

### Functional annotation of the identified significant SNPs

The online tools SNPInfo (https://snpinfo.niehs.nih.gov/) and HaploReg v 4.1 (https://pubs.broadinstitute.org/mammals/haploreg/haploreg.php) were used to predict putative functions of the identified SNPs. Expression quantitative trait loci (eQTL) analysis was performed to assess whether specific SNP might influence the mRNA expression levels of its annotated genes in whole blood cells and normal ovary tissues from the genotype-tissue expression (GTEx, V7) project (http://www.gtexportal.org/home/). To predict the potential miRNA binding with the 3′ untranslated region (3′-UTR) region of *GEN1*, we used the online tools including MirSNP (http://bioinfo.bjmu.edu.cn/mirsnp/search/), TargetScan (http://www.targetscan.org/vert_71/) and PicTar (http://pictar.mdc-berlin). The GEN1 expression levels in both normal or cancer tissues were analyzed using Sangerbox (http://vip.sangerbox.com/register.html) [14], Oncomine (https://www.oncomine.org/) and the Human Protein Atlas *(*https://www.proteinatlas.org) datasets*.* The Oncomine Database was also used to confirm the relationship between gene expression levels and clinicopathological characteristics including tumor grade, stage and tumor status. Association between gene expression levels and survival of ovarian cancer patients was performed by using online tool Kaplan–Meier plotter (http://kmplot.com/analysis/) [15].

### Cell lines and culture

Two established human ovarian cancer cell lines SKOV3 (RRID: HTB-77) and OVCA-433 (RRID: CVCL_0475) were obtained from the Cell Bank of the Chinese Academy of Science. All cells were cultured in Dulbecco’s modified Eagle’s medium (DMEM, HyClone, Thermo Scientific, USA) supplemented with 10% fetal bovine serum (Gibco, Life technologies, USA), 100 U/ml penicillin (Biowest, Nuaillé, France), and 100 U/ml streptoc-mycin (Biowest, Nuaillé, France) and incubated at 37 °C in a humidified atmosphere with 7% of CO_2_. Short tandem repeat (STR) profiling was used to authenticate all cell lines within the last 3 years. All experiments were performed with mycoplasma-free cells.

### Western blot analysis

Ovarian cancer cells were harvested, washed with cold 1 × PBS, and lysed with RIPA lysis buffer (Beyotime Institute of Biotechnology, Haimen, China) for 30 min on ice. Cell lysates were centrifuged at 12,000*g* for 15 min at 4 °C and supernatant was collected. The total protein concentration was measured by BCA Protein Assay kit (Beyotime Institute of Biotechnology). Equal amounts (30 μg per load) of protein samples were subjected to SDS‑PAGE electrophoresis and transferred on to polyvinylidene fluoride (PVDF) membranes (Millipore, Billerica, MA, USA). The blots were blocked in 8% non‑fat milk, and incubated with primary antibodies, followed by incubation with secondary antibodies conjugated with horseradish peroxidase (HRP). The protein bands were developed with the chemiluminescent reagents (Millipore). Antibody to GEN1 was from Abcam (ab198989) and antibody to β‑actin (Cat No: 66009–1-Ig) and alpha tubulin (Cat No: 66031–1-Ig) were purchased from Proteintech.

### Plasmid construction and infection

The recombinant plasmid pENTER-*GEN1* containing human full cDNA sequence of *GEN1* was purchased from Vigene Biosciences (Jinan, China). Then the cDNA sequence of *GEN1* was subcloned into lentivirus vector, which was produced by co-transfecting 293 T cells with psPAX2, pMD2.G and pCDH-puro expression vectors. Virus was harvested after 72 h by filtering the virus-containing medium through 0.45 μM Steriflip filter (Millipore). Ovarian cancer cells were infected by incubating cells with medium containing indicated virus and 8 μg/mL polybrene (Sigma) for 24 h. Established stable cell lines expressing GEN1 were constructed as above. Control cell lines were generated by infection with viruses containing the empty vector by following the same protocol.

MicroRNA-1287-5p mimic and their negative control (Ribobio, Guangzhou, China) were purchased from Ribobio (Guangzhou, China). To selectively overexpress microRNA-1287-5p, both ovarian cancer cell lines, SKOV3 and OVCA-433, were infected with microRNA mimic using Lipofectamine 3000 (Invitrogen-Life Technologies, Carlsbad, CA, United States). Control cell lines were generated by infection with plasmids containing the empty vector by following the same protocol.

### Luciferase reporter assay

The constructed Psi-CHECK2 vector carrying the 3′UTR of *GEN1* with either rs56070363 C or rs56070363 T was synthesized by Sangon Biotech (Shanghai, China). Appropriate constructed plasmids containing renilla luciferase plasmid were transfected into SKOV3 and OVCA-433 cells in 96-well plates using Lipofectamine 3000 (Invitrogen-Life Technologies, Carlsbad, CA, United States). Forty-eight hours later, we collected cell lysates and perform subsequent experiments according to technical manual of the Dual Luciferase Assay Kit (Promega, Madison, WI, USA). The luciferase activities were assessed by the measurement of absorbance obtained from a Microplate Reader (BioTek Instruments, Winooski, VT, USA). Renilla luciferase activities were used to normalize the reporter luciferase activities.

### Reverse transcription quantitative real-time polymerase chain reaction (RT-qPCR)

Total RNAs were isolated from both SKOV3 and OVCA-433 cells using the Trizol reagent (Invitrogen, Life technologies, USA) and reversely transcribed into cDNA using the PrimeScript TM RT reagent Kit (Takara Biotechnology, Shiga, Japan). The detailed information of primers was shown in Additional file 1: Table S1. Three independent experiments were performed for final analyses using the 2^−ΔΔCT^ relative quantification method.

### Cell proliferation assay

Ovarian cells with a density of 2 × 10^3^ cells per well were plated in 96-well plates with 100 μL maintenance medium. Cell Counting Kit-8 (CCK-8) (Dojindo Laboratories, Kumamoto, Japan) was applied to record cell growth at 1–7 day and the number of viable cells was assessed through measurement of absorbance at 450 nm by a Microplate Reader (BioTek Instruments, Winooski, VT, USA). The proliferation index was calculated as experimental OD value/control OD value. Cell numbers were calculated with the following equation, cell number = proliferation index × 1000.

### Colony formation assay

Ovarian cells with a density of 500 per well were seeded in 6-well plates with fresh medium added to allow cell growth for at least one week. After staining with gentian violet (Beijing Solarbio Science and Technology Co., Ltd., Beijing, China), the colonies with more than 50 cells were counted.

### Cell invasion and migration assay

To assess cellular invasion and migration, a 24-well plate with the two-chamber plate (BD Biosciences, San Jose, CA) and an 8-μm (pore size) polycarbonate filter between chambers were obtained. For the invasion assay, the upper chamber was pre-coated with Matrigel to simulate the basement membrane. SKOV3 and OVCA-433 cells, both with *GEN1* overexpression and their control cells, were seeded in the upper chamber in serum-free medium. These cells were then allowed to invade or migrate for 24 h at 37°C towards a lower chamber containing serum-supplemented medium. The cells were then fixed in 4% paraformaldehyde for 30 min and stained with crystal violet for 10 min. All cells were counted at × 200 magnification under an inverted microscope.

### Enrichment analysis

The Gene Ontology (GO), Kyoto Encyclopedia of Genes and Genomes (KEGG) enrichment analyses, and Gene Set Enrichment Analysis (GSEA) concerning on *GEN1* were conducted using the web-based tool Comprehensive Analysis on Multi-Omics of Immunotherapy in Pan-cancer (CAMOIP) database (http://www.camoip.net/) [16]. The results of these analyses were retrieved from the CAMOIP platform.

### Tumor immune microenvironment (TIME) analysis

Gene expression data were used to characterize *GEN1*-related immune microenvironment in ovarian cancer using several bioinformatics tools. The ESTIMATE (estimation of stromal and immune cells in malignant tumor tissues using expression data) algorism was used to infer the presence of non-tumor cells based on gene expression signatures. This approach involves calculating three scores using the single sample Gene Set Enrichment Analysis (ssGSEA) method: (1) stromal score, which predicts the fraction of stromal cell types in tumor tissue based on genes expression levels related to stromal tissue; (2) immune score, which estimates the infiltration of immune cells in the tumor bulk based on the genes expression levels indicative of immune cell infiltration; and (3) estimate score, a combination of the stromal and immune scores [17]. Additionally, the Immunophenotypic Score (IPS) was used to assess the immune state of the samples. IPS employs a number of markers indicative of immune response or immune toleration to quantify and visualize four distinct immunophenotypes within a tumor sample, encompassing antigen presentation, effector cells, suppressor cells and checkpoint markers [18].

To garner more comprehensive insights into the infiltration of various immune cell subtypes, we used two distinct cell type quantification methods: deconvolution-based approaches and marker gene-based approaches TIMER [19], a deconvolution-based approach, uses linear least square regression to produces a score in arbitrary units. Similarly, quanTIseq [20], another deconvolution-based method, calculates scores using constrained least square regression. Microenvironment Cell Populations-counter (MCP-counter) [21] is a marker gene-based method for quantification of nine different types of immune cells within heterogeneous tissues, particularly optimized for microarray data analysis.

The expression data for immunological regulatory molecules, checkpoint molecules and neutrophil related markers were obtained from The Cancer Genome Atlas ovarian cancer-Ovarian Serous Cystadenocarcinoma (TCGA-OV) project. We categorized the low or high-*GEN1* expression groups on median *GEN1* expression levels. Co-expressed genes showing positive or negative correlation with *GEN1,* with an adjusted *p*-value < 0.05, were considered significantly differentially expressed. The results of these analyses were visualized using heatmaps.

### Statistical analysis

All statistical analyses in this study were performed by R software (version 3.6.2). To estimate the impact of each SNP on the OS of patients, both univariate and multivariate Cox regression models were used, providing hazard ratio (HR) and 95% confidence intervals (CI). Spearman’s correlation coefficient was used to evaluate the relationships among different gene expression. The transcripts per million (TPM) format and log2 (TPM + 1) conversion were deemed as uniform unite for further research. Three independent experiments were performed for final analyses. The graphical representations were created using GraphPad Prism and Phototshop, with result expressed as standard error of the mean (SEM) with Student’s *t*-test for the differences. Kruskal–Wallis test was used to assess the correlation between gene expression and tumor stage or grade. All *P* values reported were two-sided, and a* P* value less than 0.05 were considered statistically significant**.**

## Results

### Multiple Cox regression analysis of associations between SNPs in DSB repair pathway genes and OS of EOC patients

**Fig. 1:**
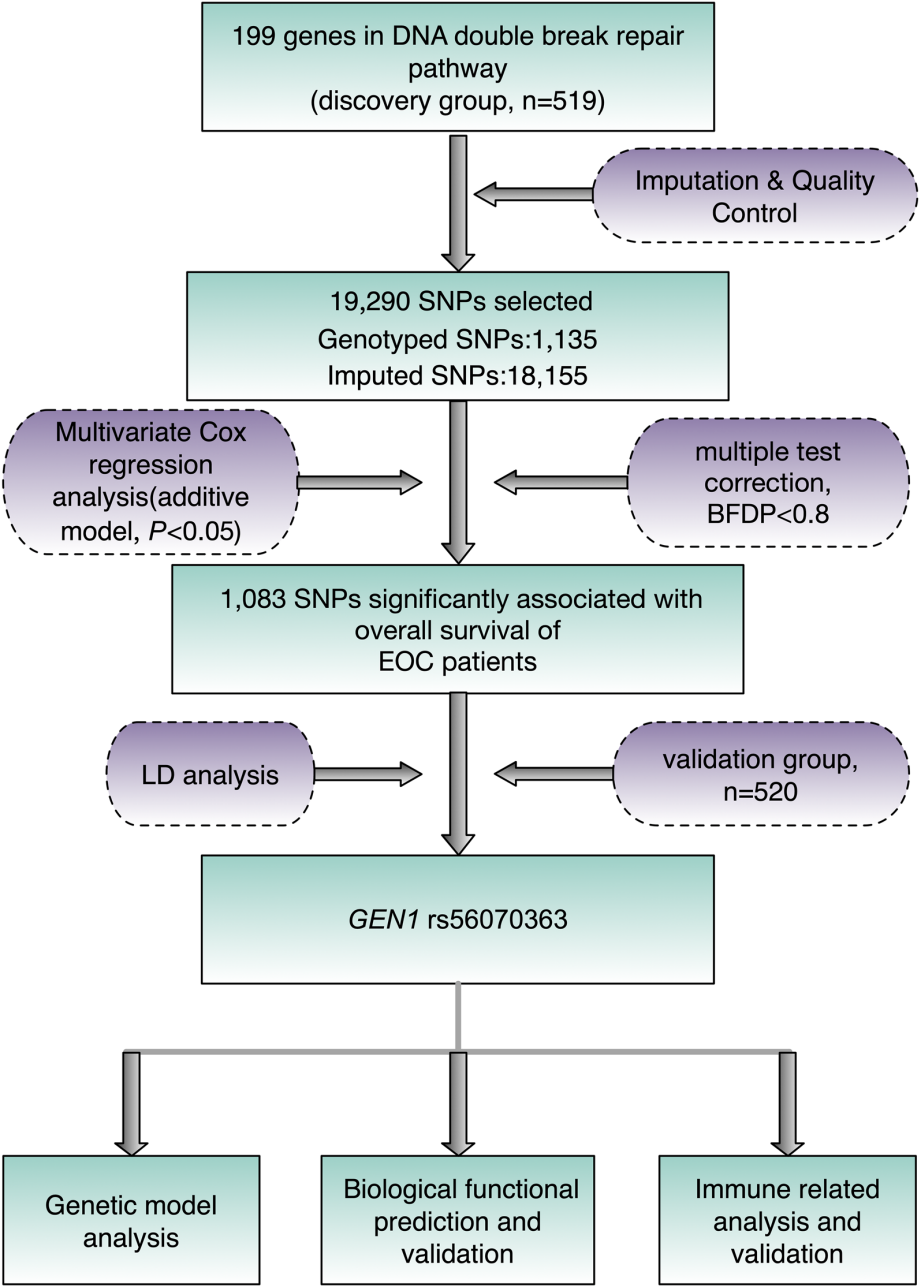
The study analysis flowchart

The study flowchart was shown in Fig. 1. Baseline characteristics of the 519 EOC patients in the discovery phase and 520 EOC patients in the validation phase were exhibited in Additional file 1: Table S2. After quality control, up to 19,290 (1,135 genotyped and 18,155 imputed) SNPs in 199 genes were available for further analysis in the discovery stage. In the single-locus analysis using an additive genetic model with adjustment for clinical variables, including age at diagnosis, stage, histology, grade, residue (optimal debulking < 1 cm), ascites and neoadjuvant chemotherapy, a total of 1,083 OS-associated SNPs were identified after multiple test correction by BFDP. However, none of these SNPs remained significant for FDR due to a high level of LD among these SNPs after imputation. These results were summarized in a Manhattan plot (Additional file 2: Fig. S1). Furthermore, we validated the most promising SNPs identified from the discovery group. Ultimately, two SNPs passed the BFDP correction in the validation stage for final analysis. Additionally, both SNPs showed significance in the meta-analysis of the two datasets with no observed heterogeneity (Table 1).

**Table 1:** SNPs of DSB repair pathway associated with survival of EOC patients

SNPs	Gene	A1	A2	MAF				
						Discovery group		
					HR (95% CI)	P*	BFDP	
rs56070363	GEN1	C	T	0.025	2.31(1.23–4.35)	0.010	0.591	
rs11893763	GEN1	T	C	0.026	2.49(1.35–4.60)	0.003	0.421	
						Validation group		
					HR (95% CI)	P*	BFDP	
rs56070363	GEN1	C	T	0.030	2.65(1.43–4.91)	0.002	0.324	
rs11893763	GEN1	T	C	0.031	2.50(1.35–4.64)	0.004	0.427	
						Combined group		
					HR (95% CI)	P*	*P*^het^	I^2^
rs56070363	GEN1	C	T	0.028	2.50(1.57–3.97)	5.95E-05	0.993	0
rs11893763	GEN1	T	C	0.028	2.48(1.54–3.98)	8.56E-05	0.766	0

SNPs, single nucleotide polymorphisms; DSB, DNA double-strand break; A1, Allele 1, the major allele; A2, Allele 2, the minor allele; EOC, epithelial ovarian carcinoma; HR, hazards ratio; CI, confidence interval; BFDP, Bayesian false-discovery probability; MAF, minor allele frequency;

P^*^, obtained in multivariate Cox regression analysis with variables including age, tumor grade, histological types, FIGO stage, residue, ascites, neoadjuvant chemotherapy and significant principal component (principal component 9 for validation group, principal component 10 for combined group, no significant principal components for discovery groups);

The results were in bold, if *P* < 0.05 or BFDP < 0.8

Then, we performed the LD analysis between these two SNPs by using haploview, and *GEN1* rs56070363 was chosen as the tag-SNP for further analysis, which was significantly associated with OS of EOC patients (adjusted HR = 2.50, 95% CI 1.57–3.97, and *P* < 0.001) in an additive genetic model in the combined dataset using stepwise multivariate Cox regression analysis (Table 2).

**Table 2:** Stepwise multivariate Cox regression analysis for the identification of significant SNPs in EOC patients

Variables	Category	Frequency	HR (95% CI)	P*
FIGO Stage	I–II/III–IV	201/790	2.54 (1.70–3.79)	5.04E−06
residue	≤ 1 cm/> 1 cm	810/103	1.63 (1.14–2.34)	0.008
Platinum response	Sensitive/resistant	548/238	3.90 (2.94–5.17)	< 2E−16
PC10			131.43 (2.04–8479.28)	0.022
*GEN1* rs56070363	CC/CT/TT	981/53/2	2.33 (1.53–3.54)	8.59E−05

The results were in bold, if *P* < 0.05

SNPs, single nucleotide polymorphisms; EOC, epithelial ovarian carcinoma; HR, hazards ratio; CI, confidence interval; FIGO, international federation of gynecology and obstetrics;

P^*^, obtained in a stepwise multivariate Cox regression analysis, and variables included age, tumor grade, histological types, FIGO stage, residue, ascites, neoadjuvant chemotherapy, platinum treatment response, PC (principal component)10 and *GEN1* rs56070363;

### Genetic associations of *GEN1* rs56070363 with EOC survival

Among all patients, the frequencies of *GEN1* rs56070363 genotype (CC, CT, TT) were 94.7%, 5.1% and 0.2% respectively, and the allele frequencies (C and T) were 97.2% and 2.8% respectively. Univariate analysis indicated a significant reduction in OS for patients with the *GEN1* rs56070363 mutant genotype (CT + TT). The Kaplan Meier survival curves of different groups were shown in Additional file 3: Fig. S2. In the multivariable analysis, adjusting for clinical variables and significant principal components, the *GEN1* rs56070363 CT + TT genotype was associated with poorer OS compared to the CC genotype (adjusted HR = 2.37, 95% CI 1.53–3.66 and *P* < 0.001, Table 3). Subsequently, we evaluated the impact of *GEN1* rs56070363 on OS using different genetic models. The results revealed a significant correlation between *GEN1* rs56070363 and OS under the dominant model, leading us to employ this model in subsequent analyses.

**Table 3:** Genetic associations of *GEN1* rs56070363 with survival of EOC patients

Genotype		Number/event	HR(95%CI)	P*
		Discovery group		
GEN1	CC	492/122	1.00	
rs56070363	CT	26/12	2.31 (1.23–4.35)	0.010
	TT	0/0	–	–
	Dominant model	26/12	2.31 (1.23–4.35)	0.010
	Recessive model	–	–	–
	Additive model	492/26/0	2.31 (1.23–4.35)	0.010
		Validation group		
GEN1	CC	489/115	1.00	
rs56070363	CT	27/12	2.99 (1.55–5.79)	0.001
	TT	2/0	–	–
	Dominant model	29/12	2.94 (1.52–5.68)	0.001
	Recessive model	516/127	–	–
	Additive model	489/27/2	2.70 (1.45–5.04)	0.002
		Combined group		
GEN1	CC	981/237	1.00	
rs56070363	CT	53/24	2.37 (1.53–3.66)	1.10E-04
	TT	2/0	–	–
	Dominant model	55/24	2.35 (1.52–3.64)	1.27E-04
	Recessive model	1034/261	–	–
	Additive model	981/53/2	2.28 (1.49–3.50)	1.62E-04

The results were in bold, if *P* < 0.05

EOC, epithelial ovarian carcinoma; HR, hazards ratio; CI, confidence interval

^*^The multivariate Cox regression analyses were adjusted for variables including age, tumor grade, histological types, FIGO stage, residue, ascites, neoadjuvant chemotherapy and significant principal component (principal component 9 for validation group, principal component 10 for combined group, no significant principal components for discovery groups)

### Stratified analysis between unfavorable genotypes and EOC survival

To optimally identify patients who would most benefit our survival prediction model, we performed stratified Cox regression analysis. This analysis aimed to pinpoint subgroups among all EOC patients who might experience prolonged survival, with adjustments made for various covariates. The results of this stratified analysis for *GEN1* rs56070363 were shown in Table 4. No interactive effects between other covariates and OS were identified, suggesting that significant associations with OS were more evident in patients with high-grade (adjusted HR = 2.04, 95% CI 1.29–3.24, *P* = 0.002), serous adenocarcinoma (adjusted HR = 2.48, 95% CI 1.56–3.94, *P* < 0.001), FIGO III-IV (adjusted HR = 2.63, 95% CI 1.66–4.17, *P* < 0.001), residual lesions ≤ 1 cm (adjusted HR = 2.57, 95% CI 1.49–4.43, *P* = 0.001), and ascites (adjusted HR = 2.75, 95% CI 1.67–4.54, *P* < 0.001).

**Table 4:** Stratified analysis for associations between unfavorable genotypes and survival of the patients in combined group

Select covariates	*GEN1* rs56070363 (Number/Event)		Overall survival		
	CC	CT&TT	HR (95% CI)	P^*^	*P*^†^
Age at diagnosis					0.691
≤ 55	496/100	29/11	2.70 (1.41–5.16)	0.003	
> 55	485/137	26/13	2.25 (1.25–4.07)	0.007	
Grade					0.502
Low	35/7	2/1	5.49 (0.34–87.88)	0.229	
High	836/210	48/21	2.04 (1.29–3.24)	0.002	
Histological types					0.194
Serous	716/172	46/21	2.48 (1.56–3.94)	1.25E-04	
Others^‡^	124/24	5/1	0.53 (0.06–5.10)	0.586	
FIGO Stage					0.436
I—II	194/28	10/1	6.82 (0.07–6.86)	0.745	
III—IV	760/202	43/22	2.63 (1.66–4.17)	3.79E-05	
Residue disease					0.265
≤ 1 cm	782/177	42/15	2.57 (1.49–4.43)	0.001	
> 1 cm	101/33	5/4	1.20 (0.37–3.96)	0.762	
Ascites					0.658
No	244/48	7/1	1.68 (0.21–13.11)	0.622	
Yes	636/156	40/19	2.75 (1.67–4.54)	7.57E-05	
Neoadjuvant					0.424
No	776/195	43/20	2.08 (1.29–3.33)	0.002	
Yes	201/42	12/4	3.50 (1.11–11.06)	0.033	
Platinum treatment response					0.214
Sensitive	524/93	33/14	2.03 (1.13–3.64)	0.018	
Resistant	231/93	13/7	3.91 (1.72–8.85)	0.001	

The results were in bold, if *P* < 0.05 (the stratified factor in each stratum excluded)

EOC, epithelial ovarian carcinoma; HR, hazards ratio; CI, confidence interval;

P^*^, *P* value of multivariate Cox regression analyses adjusted for age, tumor grade, histological types, FIGO stage, residue, ascites, neoadjuvant chemotherapy and principle components; The total number of patients was different from 1039 in some factors was due to some patients’ information was missing;

*P*^†^, *P* value of Cochran’s Q test for heterogeneity between the two groups;

^‡^other histological types include mucinous, endometrioid, clear cell and others types of EOC

### Survival ROC analysis of SNPs and OS

We used ROC curve to evaluate the sensitivity and specificity of *GEN1* rs56070363 in prognosticating the outcomes of EOC patients. First, a Cox regression model incorporating clinicopathological variables was established. Subsequently, *GEN1* rs56070363 was integrated into this model using an additive genetic model approach. ROC curves for the discovery group, validation group and a combined group were shown in Additional file 4: Fig. S3. However, we did not observe a statistically significant difference in the AUC between the two groups. The AUC of time-dependent ROC, specifically a 5-year survival timeframe for *GEN1* rs56070363, was detailed in Additional file 1: Table S3.

### The effect of *GEN1* rs56070363 C > T on the binding ability of hsa-miR-1287-5p and 3′-UTR of *GEN1*

**Fig. 2:**
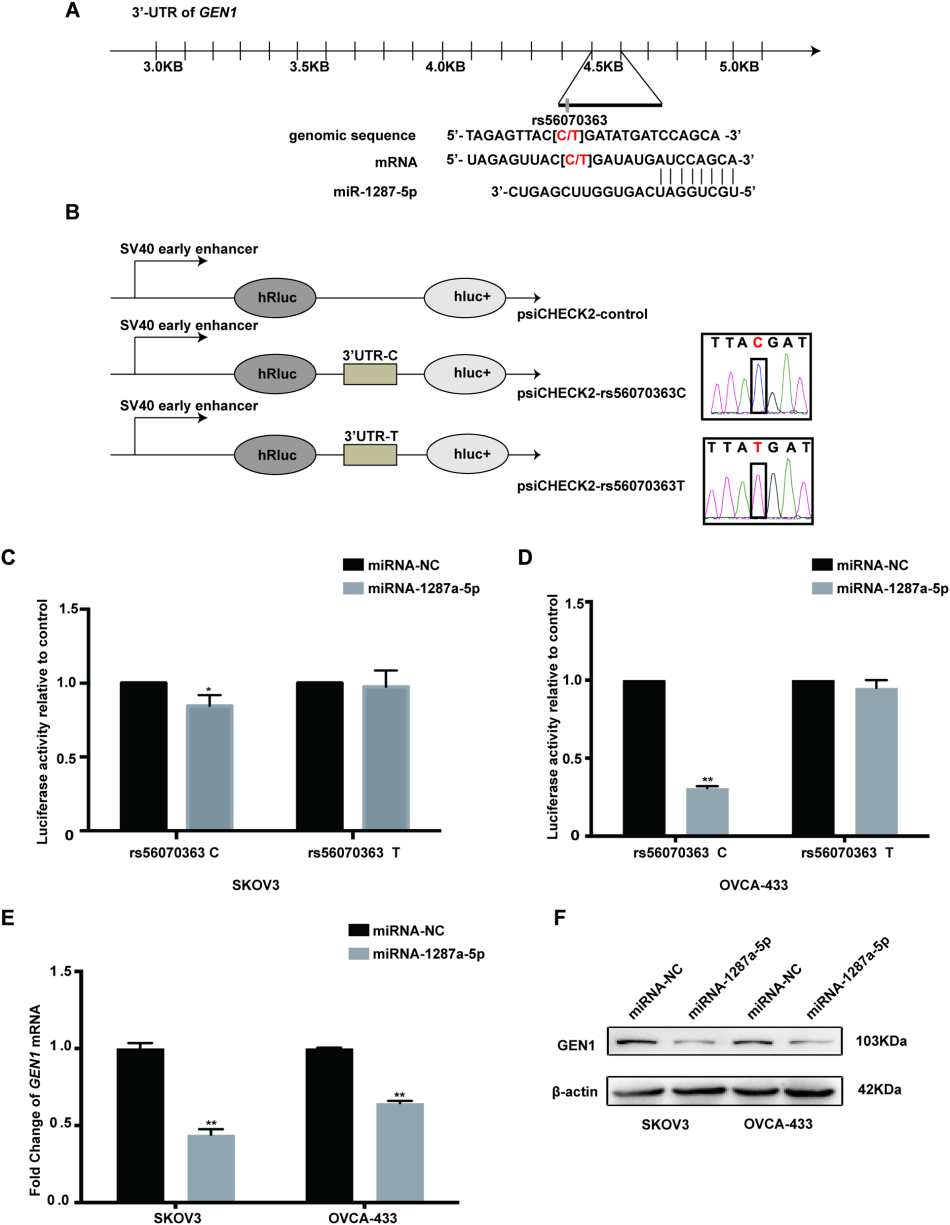
The *GEN1* rs56070363 C > T contributed to the decreased binding affinity of miRNA-1287a-5p to *GEN1* 3′-UTR. **A** Graphic representation of the detailed location of rs56070363 in the 3′UTR of *GEN1*, which was also at the miRNA-binding site with the C allele. **B** Schematic drawing of the luciferase reporter system and sequencing results of the Psi-CHECK2 vector containing rs56070363 C or T allele. **C**, **D** Luciferase activity in the presence of the miRNA-1287a-5p transfected into SKOV3 and OVAC-433 cell lines. **E** Expression of *GEN1* was detected by the qRT-PCR assay in SKOV3 and OVAC-433 cells overexpressing miRNA-1287a-5p and control cells. **F** Expression of GEN1 was detected by western blot assay in SKOV3 and OVAC-433 cells overexpressing miRNA-1287a-5p and control cells. *P < 0.05. **P < 0.01. Error bars, ± SEM from three biological replications

Utilizing the online prediction tools SNPInfo (https://snpinfo.niehs.nih.gov/) and ensemble (http://www.ensembl.org/), we found that *GEN1* rs56070363 was located at the 3 '- UTR region of *GEN1*, which was the critical binding site of microRNA. We hypothesized that the *GEN1* rs56070363 C > T change might alleviate the binding affinity of miRNA, thereby affecting *GEN1* expression and the prognosis of EOC patients. To identify specific microRNAs that interact with the *GEN1* 3 'UTR, we used online tools such as MirSNP (http://bioinfo.bjmu.edu.cn/mirsnp/search/), TargetScan (http://www.targetscan.org/vert_71/) and PicTar (http://pictar.mdc-berlin). According to these predictions, we speculated that the transition from the C to T allele at rs56070363 could weaken the binding capacity of *GEN1* 3′-UTR with hsa-miR-1287-5p (Fig. 2A). To validate this hypothesis, we constructed Psi-CHECK2 vector plasmids containing either the wild-type (*GEN1* rs56070363 C) or mutant (*GEN1* rs56070363 T) allele of the 3 '- UTR region. The sequencing results of these Psi-CHECK2 vectors were shown in Fig. 2B. The luciferase reporter assay revealed that in the presence of hsa-mir-1287-5p, there was a decreased in luciferase activity for the rs56070363 T allele compared to the C allele in two ovarian cell lines (Fig. 2C, D). Furthermore, overexpression of hsa-mir-1287-5p significantly inhibited both the mRNA and protein expression levels of GEN1 (Fig. 2E, F). Taking into account the impact of the rs56070363 C > T change on hsa-mir-1287-5p binding efficiency, we concluded that the alternation in binding affinity may lead to the upregulation of *GEN1* expression.

### Functional prediction of genetic variants of *GEN1*

**Fig. 3:**
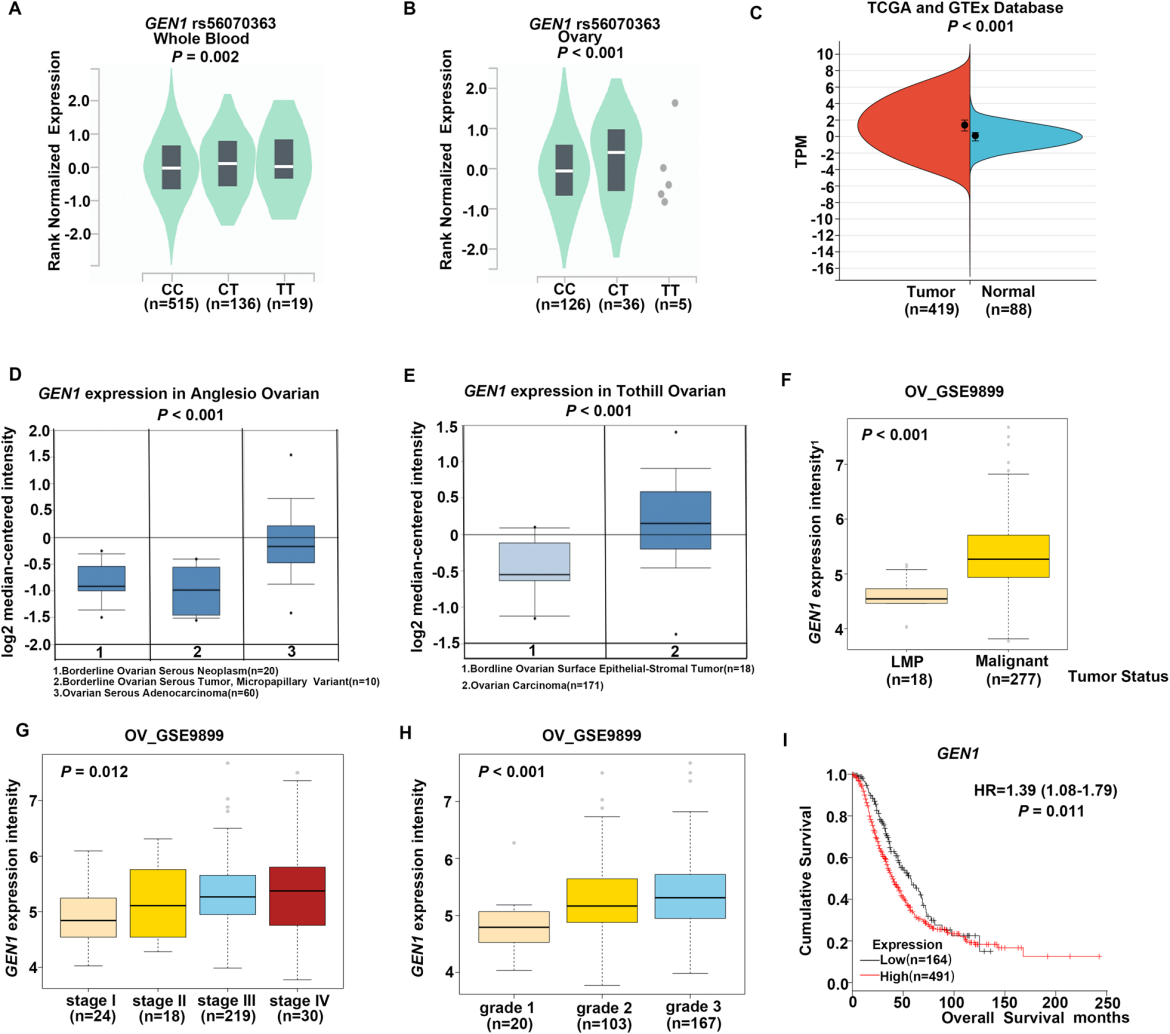
Functional prediction of genetic variants *GEN1* rs56070363. **A** The *GEN1* rs56070363 polymorphism influenced the mRNA expression of *GEN1* from GTEx database in whole blood. **B** The *GEN1* rs56070363 polymorphism influenced the mRNA expression of *GEN1* from GTEx database in ovary tissue. **C** *GEN1* expression in normal ovarian tissues (n = 88, right) and human epithelial ovarian carcinoma (n = 419, left) from TCGA database. TPM, Transcripts Per Kilobase of exon model per Million mapped reads. **D.** *GEN1* expression in Bordline Ovarian Surface Epithelial-Stromal Tumor (n = 18, left) and Ovarian Carcinoma (n = 171, right) from Tothil ovarian dataset. **E** *GEN1* expression in Borderline Ovarian Serous Neoplasm (n = 20, left), Borderline Ovarian Serous Tumor, micropapillary variant (n = 10, middle) and Ovarian Serous Adenocarcinoma (n = 60, right) from Anglesio ovarian dataset. **F** *GEN1* expression in LMP (low malignant potential, n = 18) tumor and malignant tumor (n = 277) from GSE9899 ovarian dataset. **G** The correlation between the mRNA expression of *GEN1* and tumor stage in GSE9899 ovarian dataset. **H** The correlation between the mRNA expression of *GEN1* and tumor grade in GSE9899 ovarian dataset. **I** Kaplan–Meier analyses with the log-rank test for overall survival stratified by *GEN1* mRNA expression levels

To validate the role of *GEN1* rs56070363 in regulating gene expression, we searched GTEx database, which includes mRNA expression data for *GEN1* rs56070363. The *GEN1* rs56070363 C > T change was associated with a significant increase in GEN1 mRNA expression, both in whole blood cells (*P* = 0.002, Fig. 3A) and normal ovarian tissues (*P* < 0.001, Fig. 3B).

To further support our hypothesis, we examined *GEN1* mRNA expression data from the Oncomine database (https://www.oncomine.org/) and the GEPIA online tool (http://gepia.cancer-pku.cn). The results showed an increase in expression level of *GEN1* in ovarian cancer tissues, compared with normal ovarian tissues (*P* < 0.001, Fig. 3C). Additionally, a significant difference in *GEN1* mRNA expression was observed between ovarian cancer tissues and borderline tumor tissues (Fig. 3D, E) or low malignant potential tumor tissues (*P* < 0.001, Fig. 3F). Notably, the mRNA expression level of *GEN1* was also correlated with clinicopathologic characteristics such as tumor stage (*P* = 0.012, Fig. 3G) and tumor grade (*P* < 0.001, Fig. 3H). We also initiated a comprehensive search for the expression of GEN1 protein in ovarian cancer tissues from The Human Protein Atlas datasets (Additional file 5: Fig. S4A–D). Our findings suggested that while we could hypothesize an elevated GEN1 protein expression in ovarian cancer patients, the data was insufficient for conclusive statements due to the limited number of samples. To extend our investigation, we assessed GEN1 protein expression levels of cell lines and observed that ovarian cancer cell lines exhibited higher expression level compared with normal ovarian epithelial cell line (Additional file 5: Fig. S4E).

Furthermore, we used Kaplan Meier plotter to analyze the relationship between the *GEN1* mRNA expression levels and survival outcomes in ovarian cancer patients. Consistent with our earlier findings, patients with higher *GEN1* expression levels exhibited shorter OS (*P* = 0.011, Fig. 3I).

### *GEN1* promoted cell proliferation and metastasis in ovarian cancer cells

To explore the effect of *GEN1* on ovarian cancer pathogenesis, we transiently transfected the *GEN1* cDNA plasmid into SKOV3 and OVCA-433 cell lines, which exhibited relatively low baseline expression of *GEN1*. The increased levels of GEN1 mRNA and protein levels were verified by qPCR and Western blot.

**Fig. 4:**
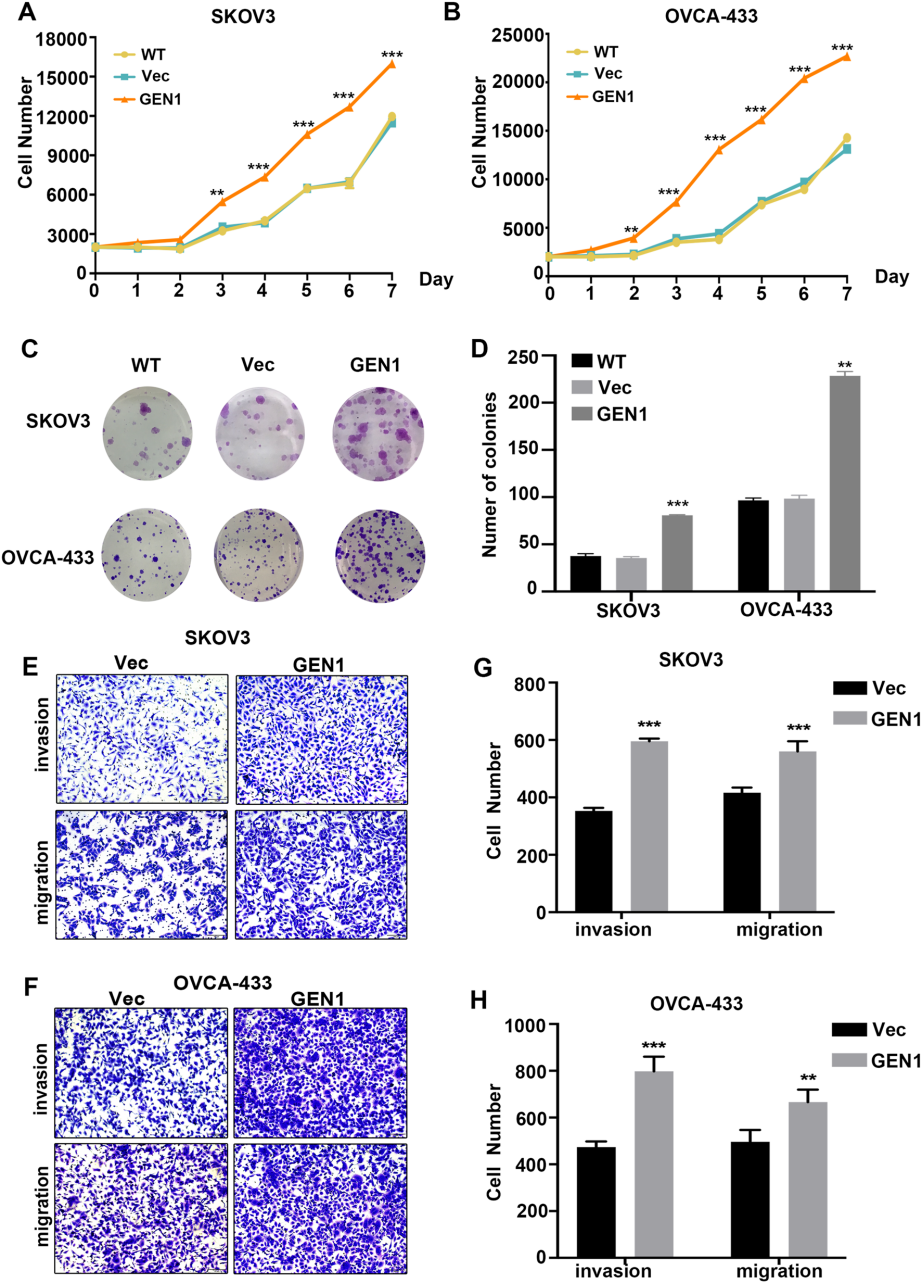
*GEN1* promoted cell proliferation and metastasis. **A**, **B** Cell viability determined by CCK8 assay in SKOV3 and OVAC-433 cell lines. **C**, **D** Representative images and number of colonies in SKOV3 and OVAC-433 cell lines. **E**, **F** Detection of cell migration and invasion by transwell assay. **G**, **H** Quantitative analysis of migration and invasion cells. Error bars, ± SEM from three biological replications. WT, wild type. **P < 0.01. ***P < 0.001

To investigate the role of GEN1 in regulating cell proliferation, we performed CCK-8 assays and colony formation assays. Compared with wild type and control cells, the overexpression of GEN1 promoted cell growth (Fig. 4A, B) in all cell lines. The colony formation assay further confirmed this finding, revealing an increase in both the number and size of colonies in cells overexpressing GEN1 (Fig. 4C, D). Additionally, we used transwell assay to explore the invasion and metastasis ability of ovarian cancer cells after GEN1 overexpression. The results showed that the ability of the migration and invasiveness was enhanced after overexpression of GEN1 in SKOV3 and OVCA-433 cell lines (Fig. 4E–H).

### Overexpression of *GEN1* may constructed an immunosuppressive environment by regulating neutrophil

**Fig. 5:**
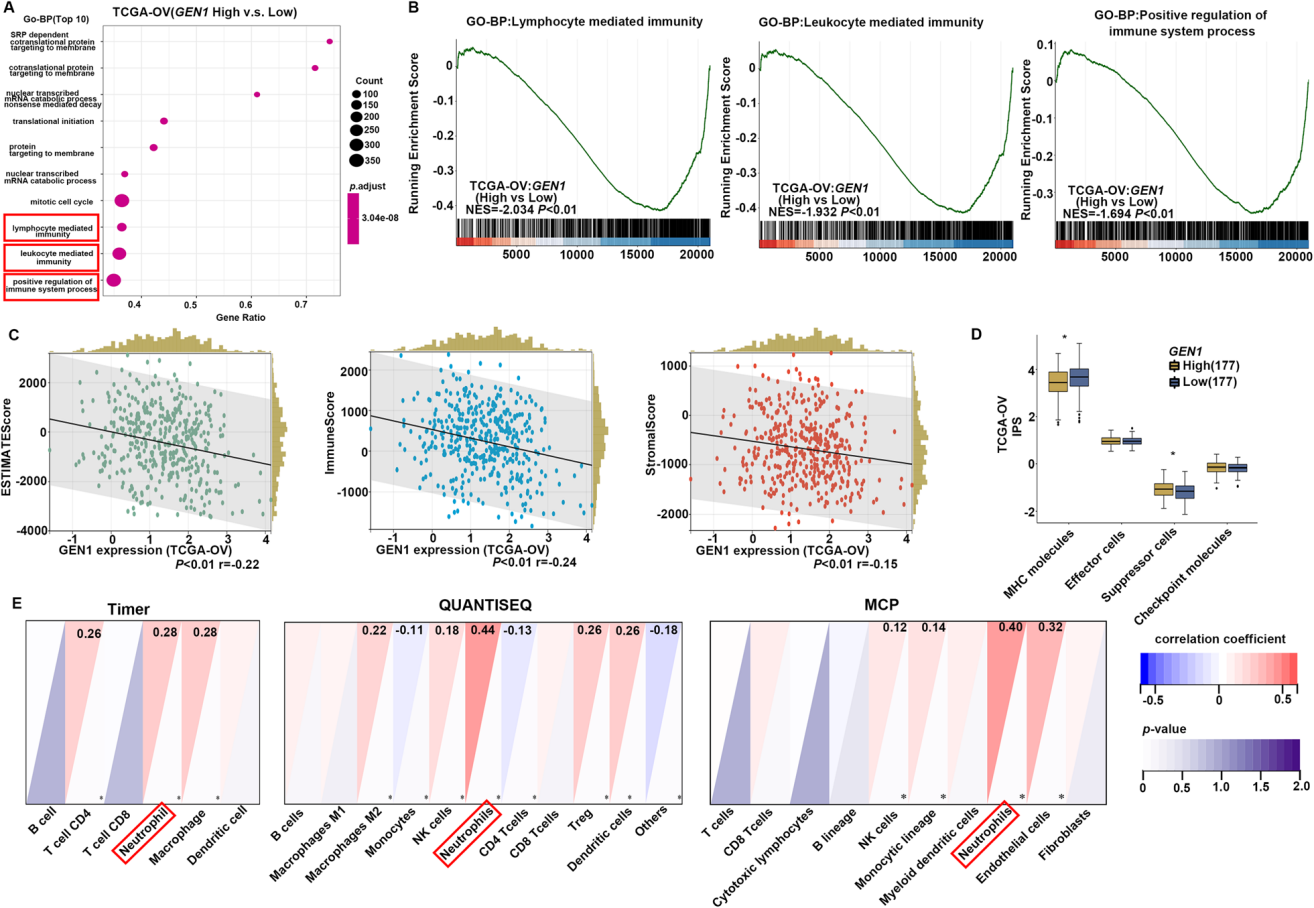
In silico analysis of *GEN1* manifest its close correlation with TIME. **A** Significantly enriched biological processes (BP) correlated with GEN1. **B** Gene set enrichment analysis (GSEA). The most involved significant hallmark correlated with *GEN1* in ovarian ancer. NES: normalized enrichment score. **C** Scatterplot showing stromal, immune and ESTIMATE (Estimation of STromal and Immune cells in MAlignant Tumor tissues using Expression data) scores for each sample in ovarian cancer. TCGA-OV, The Cancer Genome Atlas ovarian cancer- Ovarian Serous Cystadenocarcinoma. **D** Boxplot showing the comparison of antigen presentation, effector cells, suppressor cells and checkpoint scores between *GEN1* expression high and low group. **E** Correlation between *GEN1* expression and various tumor cells based on TIMER, QUANTISEQ and MCP method

The TIME is a complex ecosystem comprising tumor cells, immune cells, stromal cells, fibroblasts, extracellular matrix, and blood vessels. The intricate interplay, coexistence, and competition among these components create a unique environment that varies with the tumor type and adapt to the physiological and biochemical processes of the tumor. Dynamic change in the TIME exerts profound influence on tumor development [22]. Recent studies have shown that numerous oncogenes regulate the biological process of tumor cells by affecting the TIME. Therefore, we speculated that *GEN1* might promote the malignant phenotype of ovarian cancer cells by influencing the cellular immune process. To explore this hypothesis, we performed GO and KEGG enrichment analyses focused on *GEN1* using the CAMOIP database (http://www.camoip.net/) among ovarian cancer patients. We found that *GEN1* was involved in various immune biological process (BP, Fig. 5A). Alongside GO and KEGG analyses, Gene Set Enrichment Analysis (GSEA) analysis was also implemented to uncover potential biological functions associated with upregulated *GEN1* expression. The results indicated that increased *GEN1* expression was negatively correlated with lymphocyte mediated immunity, leukocyte mediated immunity and positive regulation of immune system process (Fig. 5B). Further investigations revealed a relationship between *GEN1* expression and various immunological regulator molecules and immune checkpoint molecules, including chemokines, receptors, MHC (major histocompatibility complex), immunostimulator and immunoinhibitor (Additional file 6: Fig. S5).

In the tumor microenvironment, immune cells and stromal cells represent the primary non-tumor components, with their proportions showing significant prognostic implications. Utilizing the ESTIMATE algorithm, we derived immune and stromal scores to quantify these components within tumors. As was shown in Fig. 5C, elevated expression of *GEN1* in ovarian cancer correlates with a reduced abundance of both stromal cells and immune cells. Further, to visualize different immunophenotypes within ovarian tumor samples, IPS scores were calculated, serving as proxies for immune activation. As expected, higher *GEN1* expression was associated with lower MHC molecules scores and higher suppressor cell scores, suggesting a dampened immune activation (*P* < 0.05, Fig. 5D). Additionally, a negative relationship between *GEN1* expression and IPS z-scores was observed, indicating reduced immunogenicity with increased *GEN1* expression level. To refine our understanding of immune cell subtype infiltration, we employed multiple quantification methods including TIMER, quanTIseq and MCP-counter. All methods concordantly indicated an enrichment in neutrophils in line with *GEN1* expression levels (*P* < 0.05, Fig. 5E).

**Fig. 6:**
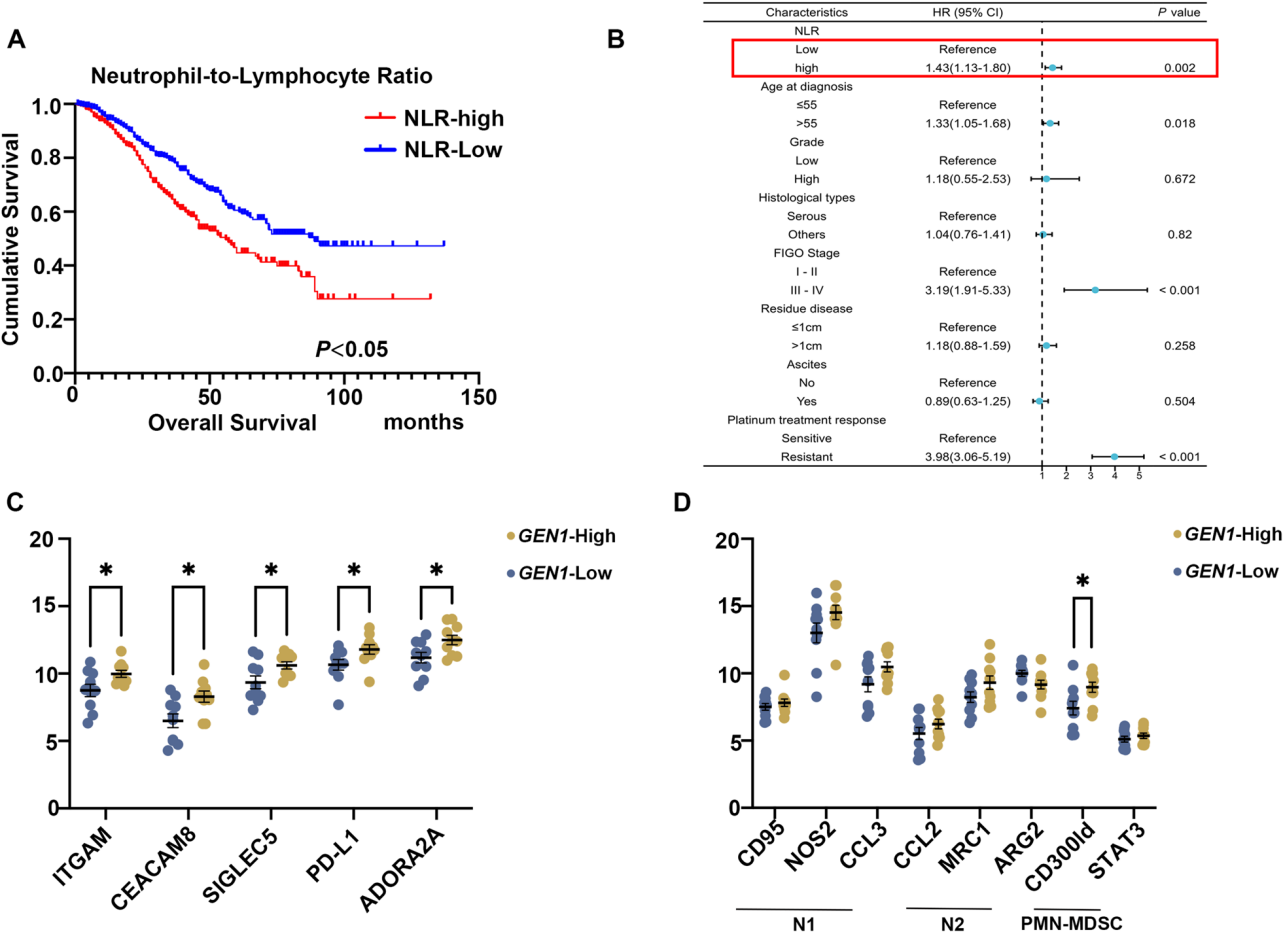
The relationship between *GEN1* expression and neutrophil. **A** Kaplan–Meier analyses with the log-rank test for OS of neutrophil-to-lymphocyte ratio in EOC patients. **B** The forest plot of multivariate analysis concerning OS. **C** Expression of neutrophil markers was detected by the qRT-PCR assay in EOC patients. **D** Expression of N1/N2 and PMN-MDSCs related markers markers was detected by the qRT-PCR assay in EOC patients. OS, overall survival. EOC, epithelial ovarian cancer

Previous studies have demonstrated that the high neutrophil-to-lymphocyte ratio (NLR) as a robust biomarker for adverse clinical outcomes in ovarian cancer. A retrospective analysis at our cancer center affirmed that a heightened NLR is a detrimental prognostic factor for OS (Fig. 6A). Moreover, in a multivariate analysis of 1036 EOC patients, an elevated NLR remained a significant variable post-adjustment for clinical parameters, as depicted in the forest plot (Fig. 6B).

To validate the predictive capability of bioinformatic approaches, we investigated the relationship between *GEN1* mRNA expression and neutrophil markers in 20 EOC patients, observing positive association with *CEACAM8(CD66b)* and *ITGAM(CD11b)* expression (Fig. 6C). Given neutrophils' dualistic nature in inflammation, capable of either anti- or pro-tumorigenic responses, we posited that *GEN1* might modulate the expression of immunosuppressive molecules, consequently hindering immune cell function and fostering an immunosuppressive TME. Indeed, *GEN1* appeared to affect the expression of *SIGLEC5*(*CD170*), *PD-L1* and *ADORA2A* (Fig. 6C).

Neutrophils can polarize into antitumorigenic phenotype (N1) and protumorigenic phenotypes (N2). However, our investigation into the impact of *GEN1* on neutrophil polarization in 20 EOC patients did not reveal significant differences in N1/N2 marker expression (Fig. 6D).

Furthermore, pathologically activated neutrophils, also known as polymorphonuclear myeloid-derived suppressor cells (PMN-MDSCs), which exhibit potent immunosuppressive activity, play pivotal roles in tumorigenesis. CD300ld is specifically expressed in normal neutrophils and is upregulated in PMN-MDSCs upon tumour-bearing. Recent studies identified CD300ld as tumor immune suppressor in the recruitment of PMN-MDSCs into tumors and its function to suppress T cell activation [23]. In our study, we found that *GEN1* expression positively correlated with the expression of the PMN-MDSCs marker CD300ld (*P* < 0.05, Fig. 6D). Above results demonstrated a potential relationship between *GEN1* expression and PMN-MDSCs which called for a further validation in the future study.

Taken together, our findings suggested that *GEN1* may promote ovarian cancer cell proliferation, invasion and migration by influencing the expression of immune inhibitory factors, thereby presumably elevating the proportion of PMN-MDSCs and constructing an immunosuppressive tumor microenvironment. Further mechanistic researches are desperately in need.

## Discussion

It is widely acknowledged that over 50% of EOC patients experience defects in homologous recombination (HR) [24] or non-homologous recombination (NHEJ) DNA repair pathways [25]. Previous studies suggested that *XRCC3* rs861539, *XRCC2* rs718282, *XRCC2* rs3218536, *BRCA1* rs1799950, *RAD51* rs1801320 and *LIG4* rs10131 were associated with the risk of ovarian cancer [26–29]. However, survival-associated genetic variants within the DSB repair pathway have not been thoroughly explored. In the present study, we randomly divided an independent GWAS dataset of Han Chinese EOC patients into two groups, conducting a two-stage analysis to evaluate the association between genetic variants in 199 DSB repair genes and patient survival. Our findings identified genetic variants *GEN1* rs56070363 and *GEN1* rs11893763 as potential biomarkers for survival prediction in EOC patients. LD analysis revealed that the *GEN1* rs56070363 C > T SNP formed a high LD block with other SNPs. Stepwise multivariate Cox regression analysis confirmed that *GEN1* rs56070363 was an independent prognostic factor for EOC patient survival. Notably, carriers of the *GEN1* rs56070363 T allele were found to have a shorter survival compared to those with the C allele. Further genotype–phenotype correlation analysis indicated an association between this variant allele and the mRNA expression level of *GEN1*. Subsequent experimental investigations unraveled the mechanism by which the *GEN1* rs56070363 C > T variant leads to poorer prognosis in patients, offering valuable insights into the molecular underpinnings of ovarian cancer prognosis.

MicroRNAs (miRNAs) represent a class of small noncoding RNAs which serve as either tumor suppressor or activators during tumorigenesis [30]. Mature miRNAs are key components in the formation of RNA-induced silencing complexes. They function to negatively regulate gene expression at the post transcriptional level, primarily by binding to complementary sequences within the 3' UTR of target mRNAs. This binding leads to the inhibition of translation or the degradation of the mRNA, thus exerting a significant influence on cellular processes related to cancer development and progression [31].

Approximately 5% of SNPs are predicted to reside in the seed region of validated miRNA binding sites, a location crucial for miRNA function [32]. Alternations in SNPs at the 3′UTR possibly affected cancer development and progression via regulating the efficiency of miRNA binding to the specific sites [33, 34]. For instance, our previous work confirmed that the impact of *RUVBL1* rs1057156 A > G variant on survival is likely attributable to a reduced binding affinity of miRNA-4294, leading to increased *RUVBL1* mRNA expression [35]. Another genetic variant, *CTNNBIP1* rs935072, is implicated in chemoresistance by altering the binding strength of miR-27a-3p to the *CTNNBIP1* 3′UTR, consequently affecting *CTNNBIP1* mRNA expression levels in epithelial ovarian cancer patients [36]. Moreover, the presence of *SET8* rs16917496 within the miR-502 mRNA seed region of the 3′UTR has been reported to influence disease risk in Chinese EOC patient, highlighting the critical role of these genomic elements in cancer pathology [37].

The *GEN1* rs56070363 variant is also located at the 3′UTR region. Utilizing in silico tools, we identified mir-1287a-5p as a novel potential regulator of *GEN1* expression*.* In ovarian cancer cell lines SKOV3 and OVCA-433, we observed a decreased luciferase activity for the rs56070363 T allele compared to the C allele in the presence of mir-1287a-5p. This reduced luciferase activity indicated a diminished binding efficiency of mir-1287a-5p, thereby influencing *GEN1* expression. Additionally, data from Oncomine and TCGA database suggested that *GEN1* may function as an oncogene, given its relatively high expression levels in cancerous ovarian tissues compared to normal and borderline cancer tissues. Overexpression of GEN1 was found to promote cell proliferation and metastasis. These findings implied that the *GEN1* rs56070363 T allele could be responsible for shorter OS and poorer prognosis of EOC patients by modulating its mRNA expression.

*GEN1* belongs to class IV of Rad2/xeroderma pigmentosum complementation group G (XPG) nuclease family [38]. Apart from NAD-dependent enzymes [39], *GEN1* constitutes a significant category of enzymes engaged in DNA damage repair process. As a dissociating enzyme in holliday junctions (HJS), *GEN1* promotes the decomposition of Holliday junctions in human cells [40–42]. Holliday junctions refer to intermediate products formed by physically connecting the sister chromatids or homologous chromosomes during homologous recombination in DNA repair [43]. Once DNA repair is completed, these persistent recombination intermediates, including HJs, must be removed by structurally selective endonucleases prior to chromosome separation in mitosis. The efficient resolution of recombination intermediates containing HJS is critical for maintaining genome stability and ensuring accurate chromosome segregation. Recent studies have primarily focused on the role of *GEN1* in breast cancer susceptibility [44] and cancer aggressiveness [45]. Unfortunately, no positive results is found except for one research from Wu Y et al. reporting that *GEN1* interference may improve the sensitivity of breast cancer cell to chemotherapy [46]. Due to limited researches, it is essential to further unveil the effect of *GEN1* in other tumors, including EOC. In our study, we found that *GEN1* contributed to the immune-suppressive function in tumor microenvironment, suggesting its importance as a therapeutic target for ovarian cancer. Further mechanistic predictions and experimental investigations have revealed its novel role in remodeling neutrophils.

Neutrophils have garnered increasing attention in cancer research due to their remarkable level of plasticity. They accumulate in tumors, responding to external stimuli, and demonstrate the capability to switch between anti- and pro-tumor phenotypes [47]. Within the tumor microenvironment, neutrophils exhibit diverse functions and are categorized using various terminologies, such as N1/N2 neutrophils, tumor-associated neutrophils, and polymorphonuclear neutrophil myeloid-derived suppressor cells (PMN-MDSCs) [48]. PMN-MDSCs are a distinct group of myeloid cells known for their immature myeloid state and immunosuppressive properties. A recent study identified CD300ld as a critical immunosuppressive molecule present on PMN-MDSCs, contributing to tumor immune evasion [23]. In our study, we observed a positive correlation between *GEN1* expression and CD300ld levels, indicating the important role of *GEN1* in modulating the functionality of neutrophils. Additionally, this finding suggested that a combined targeted therapy approach, focusing on both *GEN1* and CD300ld, could potentially enhance the efficacy and overcome resistance in immunotherapy regimens. This hypothesis warrants further investigation and validation in future studies.

However, this study has several limitations. Firstly, its retrospective nature necessitates validation through large-scale prospective studies. Secondly, the selection and identification of candidates were subject to inherent biases, notably limited geographical representation, variability in the quality of follow-up data and discrepancies in treatment adherence. In addition, the relatively small number of datasets utilized may have reduced the statistical power necessary for detecting consistent effects. Finally, further in vivo studies are required to confirm the role of *GEN1* in oncogenesis.

## Conclusions

Above all, our study offers a comprehensive analysis of the prognostic value of the *GEN1* rs56070363 SNP in DSB repair pathway. We identified that the poor overall survival was significantly associated with functional variant *GEN1* rs56070363 C > T. Further investigations revealed the mechanism underlying this association: the C > T transition results in decreased binding affinity to hsa-miR-1287-5p and subsequent upregulation of *GEN1* mRNA expression. Overexpression of *GEN1* aggregated EOC cell proliferation, invasion and migration presumably by influencing the immune inhibitory factors, thereby presumably elevating the proportion of PMN-MDSCs and then constructing an immunosuppressive tumor microenvironment. Our findings thus provide novel molecular targets and a theoretical basis for individualized treatment approaches in ovarian cancer.

### Supplementary Information

**Additional file 1: Table S1.** The primer sequence. **Table S2.** Clinical characteristics of Chinese EOC patients in discovery and validation groups. **Table S3.** The AUC values of ROC curves for predicting platinum treatment response in all the datasets.

**Additional file 2: Figure S1.** The Manhattan plot in the discovery dataset.

**Additional file 3: Figure S2.** Kaplan–Meier analyses with the log-rank for survival in EOC patients. **A** Kaplan–Meier analyses with the log-rank test for OS of *GEN1* rs56070363 in discovery group. **B** Kaplan–Meier analyses with the log-rank test for OS of *GEN1* rs56070363 in validation group. **C** Kaplan–Meier analyses with the log-rank test for OS of *GEN1* rs56070363 in combined group.

**Additional file 4: Figure S3.** Time dependent ROC curve for OS of *GEN1* rs56070363. **A** Time dependent ROC curve for OS of *GEN1* rs56070363 in discovery group. **B** Time dependent ROC curve for OS of *GEN1* rs56070363 in validation group. **C** Time dependent ROC curve for OS of *GEN1* rs56070363 in combined group.

**Additional file 5: Figure S4.** The protein expression level of GEN1 in ovarian cancer tissues and cell lines. **A**–**D** Representative images and expression level distribution of GEN1 immunohistochemical staining in ovarian cancer patients. **E** Expression level of GEN1 protein in ovarian cancer cell lines and normal ovarian epithelial cell line.

**Additional file 6: Figure S5.** Correlation between expression of immune process associated markers and *GEN1*. **A** The heatmap showed correlation between expression of immunological regulatory molecules and *GEN1*. **B** The heatmap showed correlation between expression of immune checkpoint molecules and *GEN1*. (*P < 0.05, **P < 0.01, ***P < 0.001).

## Data Availability

The data that support the findings of this study are available from the corresponding author upon reasonable request.

## Abbreviations

GWAS: Genome-wide association study
SNPs: Single nucleotide polymorphisms
DDR: DNA damage repair
DSB: DNA double-strand break
EOC: Epithelial ovarian carcinoma
HR: Hazard ratio
CI: Confidence interval
BFDP: Bayesian false-discovery probability
MAF: Minor allele frequency
FUSCC: Fudan University Shanghai Cancer Center
FIGO: International Federation of Gynecology and Obstetrics
QC: Quality control
PCA: Principal component analysis
LD: Linkage disequilibrium
ROC: Receiver operating characteristic
AUC: Area under the curve
eQTL: Expression quantitative trait loci
SEM: Standard error of the mean
TPM: Transcripts per million
GO: Gene Ontology
KEGG: Kyoto Encyclopedia of Genes and Genomes
GSEA: Gene Set Enrichment Analysis
CAMOIP: Comprehensive Analysis on Multi-Omics of Immunotherapy in Pan-cancer
IPS: Immunophenotypic Score
MCP-counter: Microenvironment Cell Populations-counter
TCGA-OV: The Cancer Genome Atlas ovarian cancer- Ovarian Serous Cystadenocarcinoma
TIME: Tumor immune microenvironment
PMN-MDSCs: Polymorphonuclear myeloid-derived suppressor cells
N1: Antitumorigenic phenotype
N2: Protumorigenic phenotype
NLR: Neutrophil-to-lymphocyte ratio
MHC: Major histocompatibility complex
STR: Short tandem repeat
