# Supplementary material for: Functional genetic variants of GEN1 predict overall survival of Chinese epithelial ovarian cancer patients
Source: J Transl Med. 2024 Jun 18;22:577. doi: 10.1186/s12967-024-05236-1 (PMC11184878; doi:10.1186/s12967-024-05236-1)
Supplement: Supplementary file 1 — Additional file 1: Table S1. The primer sequence. Table S2. Clinical characteristics of Chinese EOC patients in discovery and validation groups. Table S3. The AUC values of ROC curves for predicting platinum treatment response in all the datasets. [file 12967_2024_5236_MOESM1_ESM.docx]

**Table S1**. The primer sequence.

| **Gene** | **Forward primer** | **Reverse primer** |
| --- | --- | --- |
| *GAPDH* | 5'-GGAGCGAGATCCCTCCAAAAT-3' | 5'-GGCTGTTGTCATACTTCTCATGG-3' |
| *GEN1* | 5'-TCCCCTTGCGTAATCTTGGTG-3' | 5'-TCCAGAAGACCCATACCGAGA-3' |
| *ITGAM* | 5'-ACTGGTGAAGCCAATAACGCA-3' | 5'-TCCGTGATGACAACTAGGATCTT-3' |
| *CEACAM8* | 5'-TGCTCAGCTCACTATTGAAGC-3' | 5'-CCTATAATTCGACGGTTGGCAT-3' |
| *SIGLEC5* | 5'-GCTGCAAGGGAGATCGAACC-3' | 5'-AGGCACAGACAGATACAGAGC-3' |
| *PD-L1* | 5'-TGGCATTTGCTGAACGCATTT-3' | 5'-TGCAGCCAGGTCTAATTGTTTT-3' |
| *ADORA2A* | 5'-CGCTCCGGTACAATGGCTT-3' | 5'-TTGTTCCAACCTAGCATGGGA-3' |
| *CD95* | 5'-TCTGGTTCTTACGTCTGTTGC-3' | 5'-CTGTGCAGTCCCTAGCTTTCC-3' |
| *NOS2* | 5'-TTCAGTATCACAACCTCAGCAAG-3' | 5'-TGGACCTGCAAGTTAAAATCCC-3' |
| *CCL3* | 5'-AGTTCTCTGCATCACTTGCTG-3' | 5'-CGGCTTCGCTTGGTTAGGAA-3' |
| *CCL2* | 5'-CAGCCAGATGCAATCAATGCC-3' | 5'-TGGAATCCTGAACCCACTTCT-3' |
| *MRC1* | 5'-TCCGGGTGCTGTTCTCCTA-3' | 5'-CCAGTCTGTTTTTGATGGCACT-3' |
| *ARG2* | 5'-ACCTGATAGTGAATCCACGCT-3' | 5'-CATGGGCATCAACCCAGAC-3' |
| *CD300ld* | 5'-TCCCAGGTTACTCCATTGCC-3' | 5'-GCCTGAGCCATAAGCACACT-3' |
| *STAT3* | 5'-CAGCAGCTTGACACACGGTA-3' | 5'-AAACACCAAAGTGGCATGTGA-3' |

| Table S2. Clinical characteristics of Chinese EOC patients in discovery and validation groups. | | | |
| --- | --- | --- | --- |
| Variables | **Discovery group**  **No. (%)** | **Validation group**  **No. (%)** | ***P*^#^** |
| Age at diagnosis |  |  | 0.515 |
| Median (range) | 55 (21-80) | 56 (20-88) |  |
| Grade |  |  | 0.555 |
| Low | 17 (3.3) | 20 (3.8) |  |
| High | 439 (84.6) | 447 (86.0) |  |
| Not available | 63 (12.1) | 53 (10.2) |  |
| Histology |  |  | 0.672 |
| Serous | 374 (72.1) | 389 (74.8) |  |
| Other* | 70 (13.4) | 61 (11.7) |  |
| Not available | 75 (14.5) | 70 (13.5) |  |
| FIGO Stage |  |  | 0.179 |
| I-II | 110 (21.2) | 95 (18.3) |  |
| III-IV | 391 (75.3) | 414 (79.6) |  |
| Not available | 18 (3.5) | 11 (2.1) |  |
| Residue disease |  |  | 0.802 |
| ≤1cm | 411 (79.2) | 415 (79.8) |  |
| >1cm | 56 (10.8) | 50 (9.6) |  |
| Not available | 52 (10.0) | 55 (10.6) |  |
| Ascites |  |  | 0.875 |
| No | 125 (24.1) | 127 (24.4) |  |
| Yes | 337 (64.9) | 341 (65.6) |  |
| Not available | 57 (11.0) | 52 (10.0) |  |
| Neoadjuvant |  |  | 0.234 |
| No | 399 (76.9) | 422 (81.2) |  |
| Yes | 118 (22.7) | 96 (18.5) |  |
| Not available | 2 (0.4) | 2 (0.4) |  |
| Platinum treatment response |  |  | 0.771 |
| Sensitive | 282 (54.3) | 277 (53.3) |  |
| Resistant | 117 (22.5) | 127 (24.4) |  |
| Not available | 120 (23.1) | 176 (22.3) |  |
| Abbreviations: EOC, epithelial ovarian carcinoma; FIGO, international federation of gynecology and obstetrics;  *P*^#^, *P* value obtained in Chi-square test;  *-- other histology include mucinous, endometrioid, clear cell and others types of EOC. | | | |

| **Table S3.** The AUC values of ROC curves for predicting platinum treatment response in all the datasets | | | | | | |  |
| --- | --- | --- | --- | --- | --- | --- | --- |
| **Variables** | **Discovery group** | | **Validation group** | | **Combined group** | |  |
|  | **AUC (95% CI)** | ***P*^#^** | **AUC (95% CI)** | ***P*^#^** | **AUC (95% CI)** | ***P*^#^** | |
| Clinical variables | 0.64 (0.55-0.73) |  | 0.75 (0.67-0.83) |  | 0.74 (0.66-0.83) |  | |
| Clinical variables *+* *GEN1* rs56070363 | 0.64 (0.55-0.73) | 0.799 | 0.69 (0.63-0.74) | 0.844 | 0.70 (0.64-0.76) | 0.871 | |
| Abbreviations: AUC, area under curve; ROC, receiver operating characteristic; CI, confidence interval;  *P*^#^, *P* value obtained by comparing between ROC curves incorporating only clinical factors and ROC curves incorporating *GEN1* rs56070363 and clinical factors;  The results were in bold, if *P*<0.05. | | | | | | | |
