# Supplementary figures and images for: Functional genetic variants of GEN1 predict overall survival of Chinese epithelial ovarian cancer patients
Source: J Transl Med. 2024 Jun 18;22:577. doi: 10.1186/s12967-024-05236-1 (PMC11184878; doi:10.1186/s12967-024-05236-1)

Manhattan Plot in discovery group

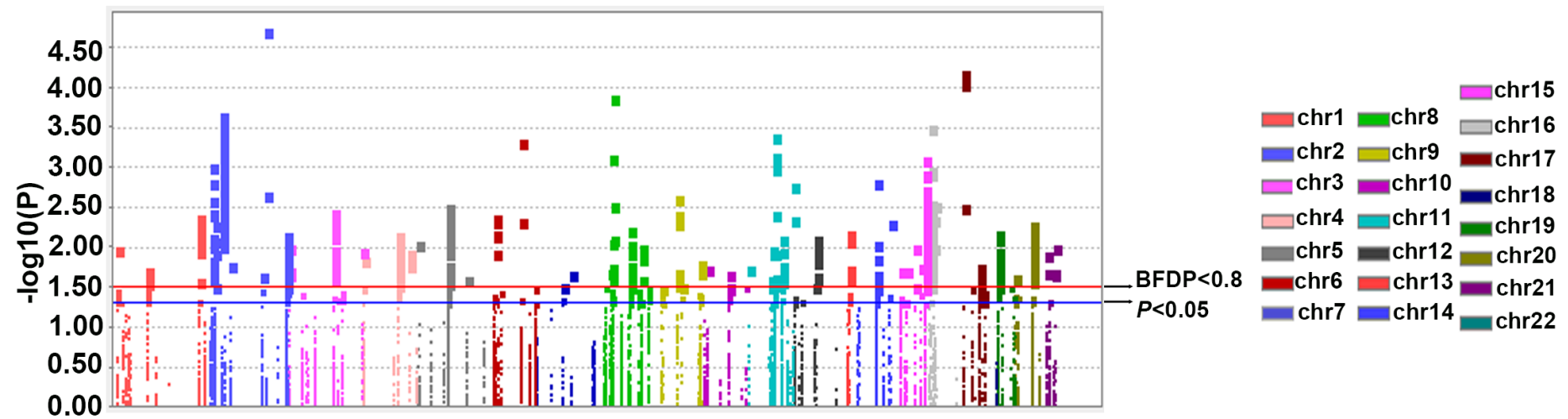

Supplement: Supplementary file 2 — Additional file 2: Figure S1. The Manhattan plot in the discovery dataset. [file 12967_2024_5236_MOESM2_ESM.pdf]

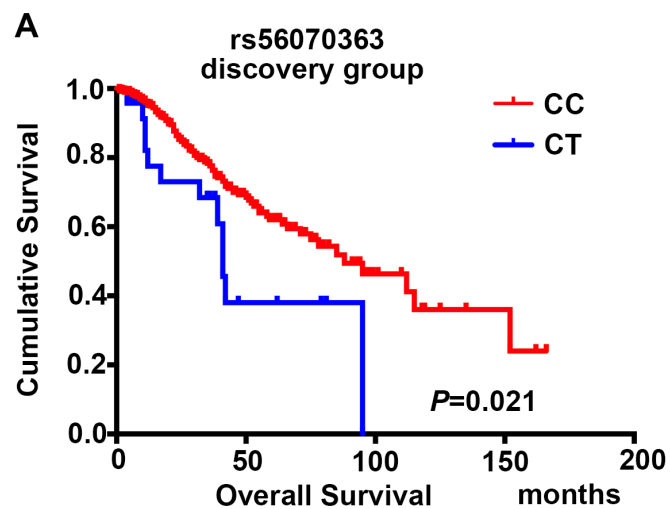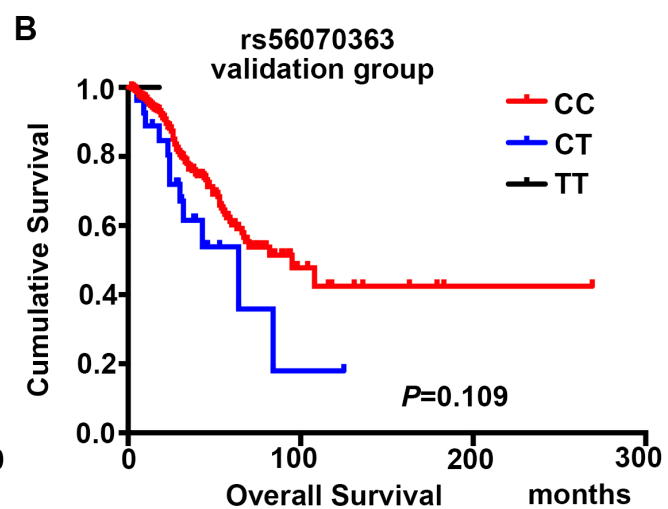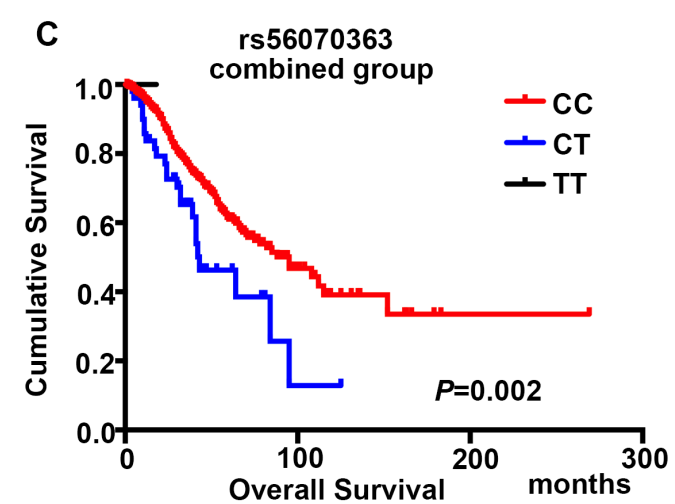

Supplement: Supplementary file 3 — Additional file 3: Figure S2. Kaplan–Meier analyses with the log-rank for survival in EOC patients. A Kaplan–Meier analyses with the log-rank test for OS of GEN1 rs56070363 in discovery group. B Kaplan–Meier analyses with the log-rank test for OS of GEN1 rs56070363 in validation group. C Kaplan–Meier analyses with the log-rank test for OS of GEN1 rs56070363 in combined group. [file 12967_2024_5236_MOESM3_ESM.pdf]

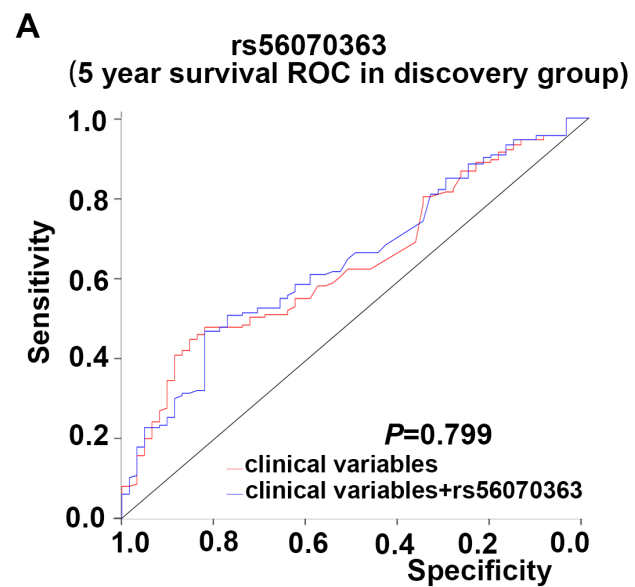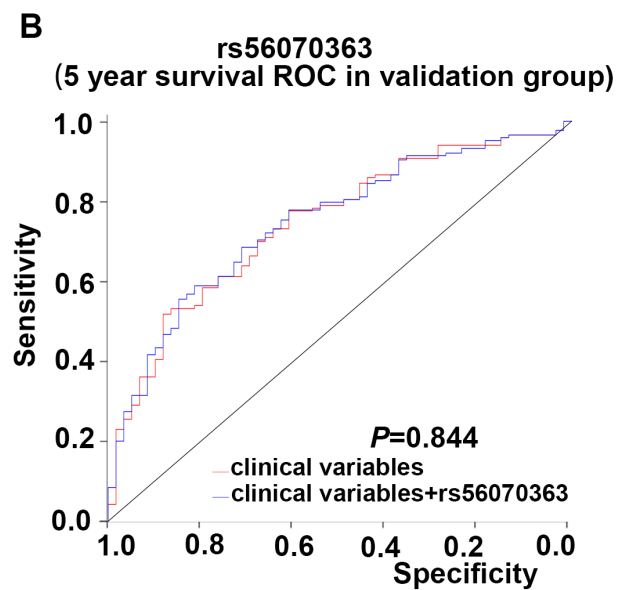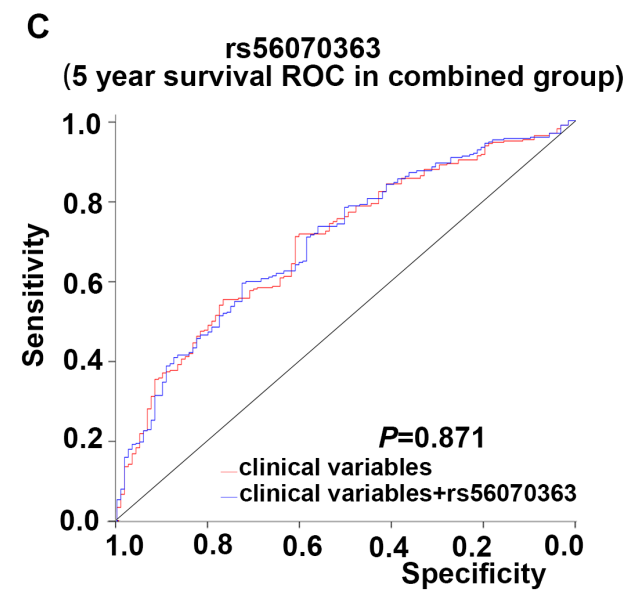

Supplement: Supplementary file 4 — Additional file 4: Figure S3. Time dependent ROC curve for OS of GEN1 rs56070363. A Time dependent ROC curve for OS of GEN1 rs56070363 in discovery group. B Time dependent ROC curve for OS of GEN1 rs56070363 in validation group. C Time dependent ROC curve for OS of GEN1 rs56070363 in combined group. [file 12967_2024_5236_MOESM4_ESM.pdf]

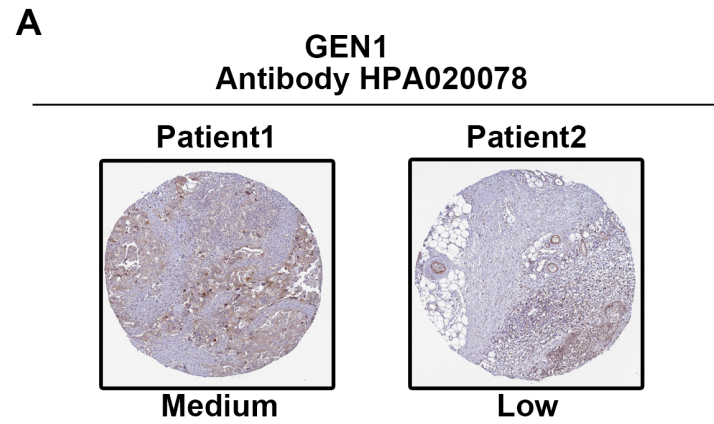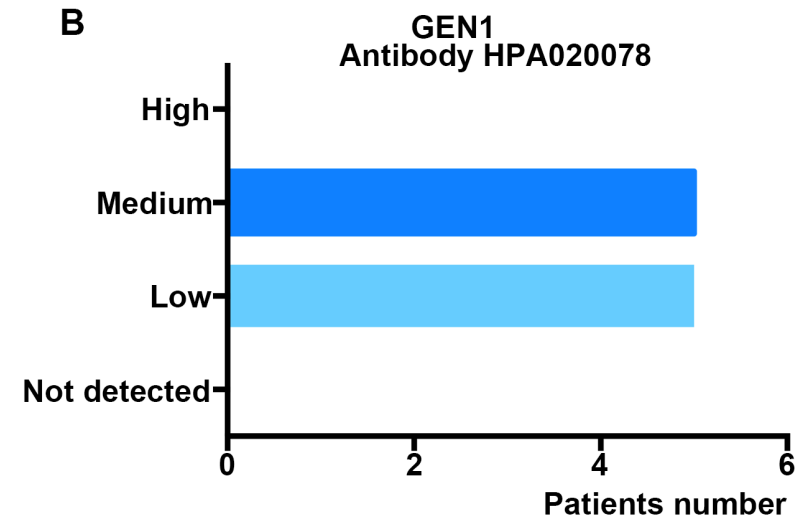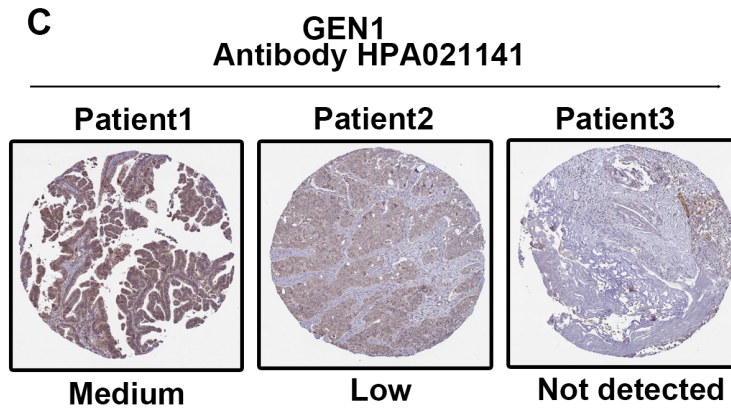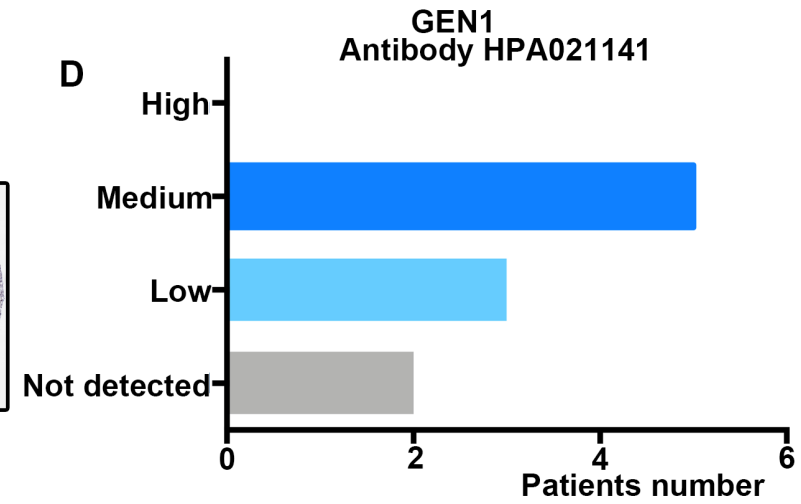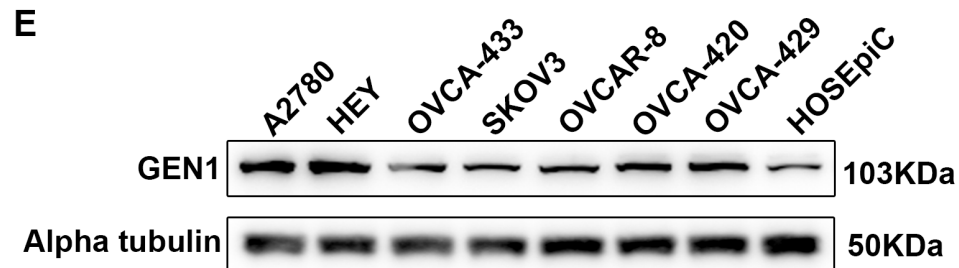

Supplement: Supplementary file 5 — Additional file 5: Figure S4. The protein expression level of GEN1 in ovarian cancer tissues and cell lines. A–D Representative images and expression level distribution of GEN1 immunohistochemical staining in ovarian cancer patients. E Expression level of GEN1 protein in ovarian cancer cell lines and normal ovarian epithelial cell line. [file 12967_2024_5236_MOESM5_ESM.pdf]

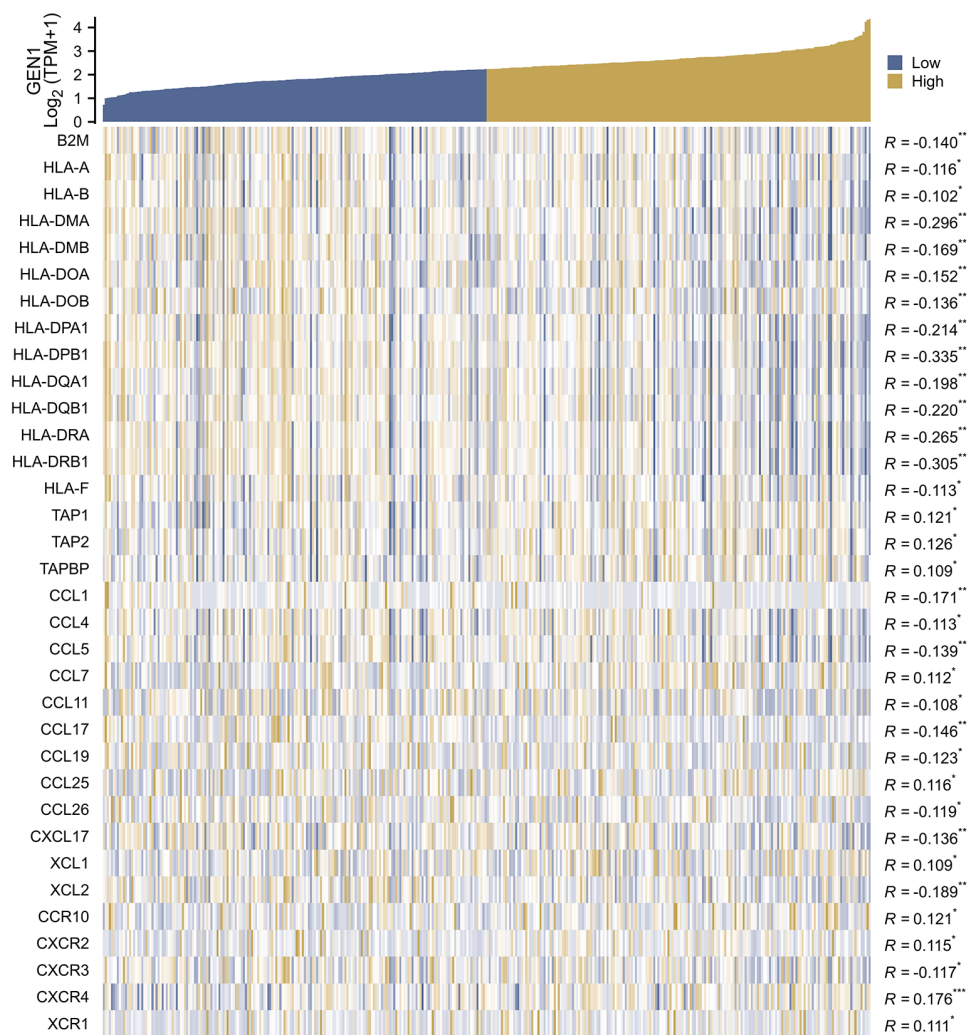

Immunological Regulatory Molecules

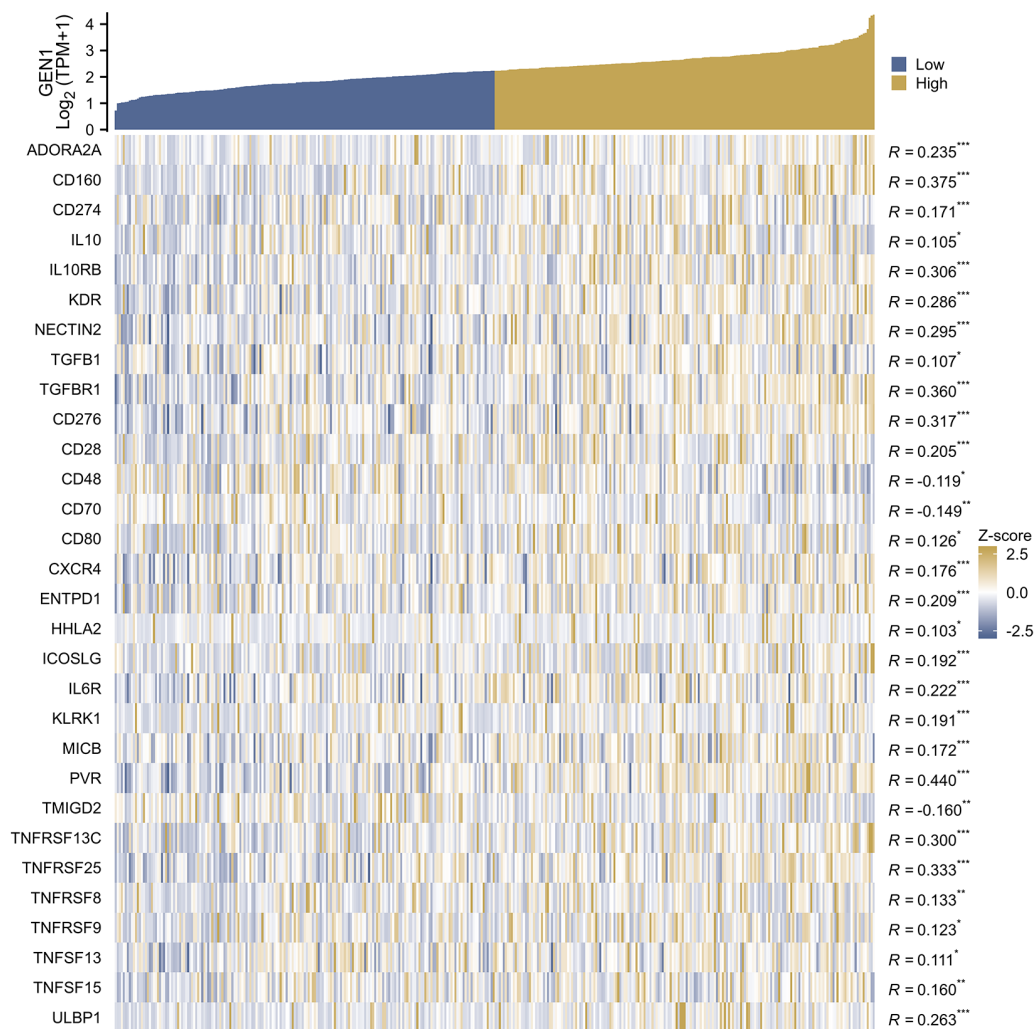

Immune Checkpoint Molecules

Supplement: Supplementary file 6 — Additional file 6: Figure S5. Correlation between expression of immune process associated markers and GEN1. A The heatmap showed correlation between expression of immunological regulatory molecules and GEN1. B The heatmap showed correlation between expression of immune checkpoint molecules and GEN1. (*P < 0.05, **P < 0.01, ***P < 0.001). [file 12967_2024_5236_MOESM6_ESM.pdf]
